# Contamination of Urban Stormwater Pond Sediments: A Study of 259 Legacy and Contemporary Organic Substances

**DOI:** 10.1021/acs.est.0c07782

**Published:** 2021-02-19

**Authors:** Kelsey Flanagan, Godecke-Tobias Blecken, Heléne Österlund, Kerstin Nordqvist, Maria Viklander

**Affiliations:** Urban Water Engineering, Department of Civil, Environmental and Natural Resources Engineering, Luleå University of Technology, Luleå 971 87, Sweden

## Abstract

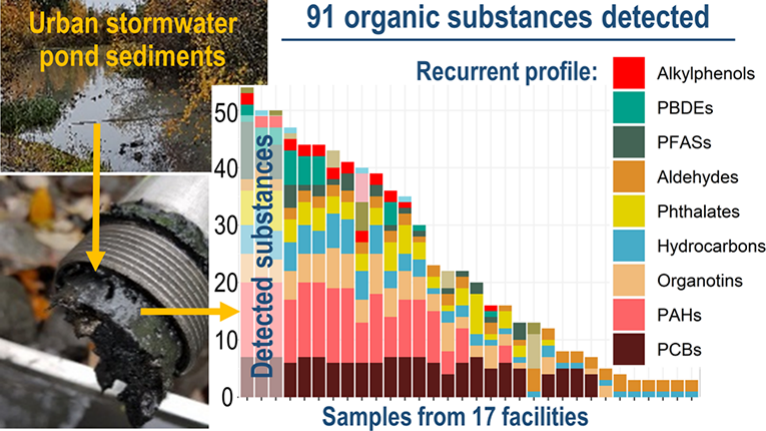

Stormwater ponds improve water quality by facilitating the sedimentation of particles and particulate contaminants from urban runoff. Over time, this function entails the accumulation of contaminated sediments, which must be removed periodically to maintain a pond’s hydraulic and treatment capacity. In this study, sediments from 17 stormwater sedimentation facilities from four Swedish municipalities were analyzed for 259 organic substances likely to be found in the urban environment. A total of 92 substances were detected in at least one sample, while as many as 52 substances were detected in a single sample. A typical profile of urban contamination was identified, including polychlorinated biphenyls, polycyclic aromatic hydrocarbons, organotins, aliphatic hydrocarbons, phthalates, aldehydes, polybrominated diphenyl ethers, perfluorinated substances, and alkylphenols. However, levels of contamination varied greatly between ponds, influenced heavily by the dilution of urban pollutants and wear particles from other sources of particles such as eroded soil, sand, or natural organic matter. For 22 of 32 samples, the observed concentrations of at least one organic substance exceeded the regulatory threshold values derived from toxicity data for both sediment and soil.

## Introduction

Urban stormwater is a vector for contamination from various anthropogenic sources^1,2^ that can degrade the quality of receiving waters.^3^ While it has long been known that urban stormwater contains suspended solids, nutrients, trace metals, chlorides, aliphatic hydrocarbons, and polycyclic aromatic hydrocarbons (PAHs),^4,5^ a growing body of research has also demonstrated the presence of a wider range of organic substances such as alkylphenols, phthalates, polybrominated diphenyl ethers (PBDEs), organotins, pesticides, and polychlorinated biphenyls (PCBs).^6−9^ Several of these substances have been prioritized by regulations such as the United States Clean Water Act^10^ and the European Union Water Framework Directive.^11^

Stormwater control measures (SCMs) have been developed to manage urban stormwater and its pollution. Many SCMs are nature-based solutions designed not only to regulate flows and improve water quality but also to contribute to urban biodiversity and amenity.^12^ Stormwater ponds are some of the most common SCMs, with tens of thousands of facilities implemented across the world since their introduction in the 1960s.^13^ In these systems, stormwater flows are regulated and the water quality is improved mainly through particle sedimentation.^14^ This treatment process is likely to affect many organic substances that are predominantly associated with the particulate phase in stormwater, including PAHs, PCBs, PBDEs, organotins, and some phthalates.^6,15,16^

Because sedimentation essentially transfers a wide range of substances from the water compartment to the sediment compartment, this contaminated sediment presents a potential environmental risk, especially during its removal and disposal. Periodic removal of sediments is a maintenance activity essential to ensuring the adequate long-term performance of stormwater ponds,^13^ and life cycle assessment has shown the management of solid waste produced by nature-based SCMs to be critical to the overall environmental impacts of these facilities.^17^ In addition, sediment contamination may represent a conflict between the wildlife habitat and water treatment functions of stormwater ponds.^18^

Indeed, bioassays of sediments collected from stormwater ponds have shown them to cause a toxic response in various organisms, including bacteria and freshwater and benthic invertebrates and amphibians,^19−23^ and many previous studies have confirmed their contamination by substances typically associated with urban runoff, such as trace metals,^24−28^ hydrocarbons,^29−31^ and PAHs.^22,31−40^ A handful of studies have also shown that stormwater pond sediments can be contaminated by historic contaminants, such as organochlorine pesticides^35,38,41,42^ and PCBs,^38,43^ contemporary pesticides,^41,42,44^ and contaminants of emerging concern, including PBDEs,^41,42,44^ alkylphenols,^42,44^ phthalates,^44^ and perfluorinated substances (PFASs).^44^

Building on this knowledge toward an understanding of the factors influencing the occurrence and extent of contamination by different substances and prioritization of substances in different contexts requires large-scale studies in which a wide range of substances are analyzed in sediments from a large number of facilities. The objective of the present study is to respond to this need by analyzing 259 organic substances in sediments from 17 stormwater sedimentation facilities (16 ponds and 1 subsurface sedimentation basin). The studied substances include aliphatic and aromatic hydrocarbons, PAHs, PCBs, alkylphenols, phthalates, brominated flame retardants (including PBDEs), PFASs, organotins, aldehydes, monocyclic aromatic hydrocarbons, methyl *tert*-butyl ether (MTBE), chlorobenzenes, chlorinated aliphatics, chlorophenols, and both historic and contemporary pesticides. Thus, this study provides the most comprehensive characterization of organic substances in stormwater pond sediments to date, addressing both legacy and contemporary contaminants likely to be present in the urban environment.

## Materials and Methods

### Presentation of Study Sites

Sediments were collected from a total of 17 stormwater sedimentation facilities (see Table S1 in the Supporting Information for facility characteristics), all designed with an objective of improving water quality, though the exact design criteria likely vary due to an absence of national guidance for a stormwater pond design. A range of typical catchment types are represented: nine facilities collect water from primarily industrial and/or commercial catchments, five collect water from mainly residential catchments, and three from roads or highways.

The facilities are located in the municipalities of Örebro (six ponds), Östersund (one pond), Stockholm (five ponds and one sedimentation tank), and Växjö (four ponds). All of these municipalities are subject to cold climates,^45^ where particle production during winter months is influenced by both studded tires and winter road maintenance practices (application of salt and gravel to improve traction).^46^ Ponds in Örebro, Östersund, and Växjö were chosen from a list of 25 previously studied ponds,^24^ prioritizing ponds with sediments consisting of relatively high percentages of fine particles (clay and silt) and metal contents exceeding Swedish guidelines for contaminated sites.

The facilities were constructed between 1988 and 2010. The facility surface areas vary from 0.006 to 1.78 hectares, with average depths between 0.73 and 2.02 m. Catchment surface areas ranged from 1.1 to 1490 hectares, leading to facility-to-catchment ratios between 0.0071 and 2.6%.

### Sediment Sampling

Sampling took place from October to December 2019. Generally, sediments were collected from two locations (inlet and outlet) in each facility using a Kajak sediment core sampler (KC Denmark) lined with a stainless steel tube and equipped with a 2 m shaft. When facilities had two inlets, sediments were collected from both in proportion to the size of the inlets and combined. In two facilities, it was not possible to collect sediments from the outlet; therefore, a total of 17 inlet samples and 15 outlet samples were collected. Around 3 L of sediments was required for analysis of all compounds; therefore, several cores were combined to obtain a composite sample of each location. As the site mean core depths varied between 6 and 45 cm depending on the site, between 4 and 22 cores were collected per site. Entire cores were placed in a stainless steel tray, homogenized using a stainless steel spoon, and divided into nine quality controlled glass jars for different analyses. This sampling strategy implies that the observed concentrations are essentially the mean samples integrated over the time the sediments have accumulated, which vary between ponds as a function of their date of construction or most recent sediment removal.

When possible, the composite samples were divided by quartering. However, some samples were too liquid to be quartered; in this case, they were spooned into each jar, alternating between jars and mixing between spoonfuls. All equipment in contact with the samples was rinsed three times in water from the facility before sampling. Equipment blanks were carried out for all substances consistently quantified in the sediment (at most two samples with concentrations below the limit of quantification) to ensure that there was no systematic contamination during sampling.

### Sediment Analysis

The list of analyzed substances was selected to include organic substances identified by previous studies as priority pollutants in urban^47,48^ or road^49^ runoff, as well as priority substances from the European Union Water Framework Directive previously quantified in studies of urban stormwater^6,16,50^ or urban soil.^51^ Samples were submitted for analysis to an accredited laboratory (ALS Scandinavia), where they were analyzed without prior sieving. Table 1 presents a list of all studied substances, analytical methods, and limit of quantification (LOQ). For some substances (brominated flame retardants, alkylphenols, and phthalates), LOQs varied between samples due to matrix effects, so the range of LOQs is presented.

**Table 1:** List of Analyzed substance’s Names and Abbreviations, Analytical Methods, and LOQ

substance family	analysis method	standard(s)	substances (abbreviation if applicable, limit of quantification—LOQ—in μg/kg dry mass)
*hydrocarbons*	GC–MS	SPIMFAB	C_5_–C_8_ aliphatics (10000), C_8_–C_10_ aliphatics (10000), C_10_–C_12_ aliphatics (10000), C_12_–C_16_ aliphatics (10000), C_16_–C_35_ aliphatics (10000), C_8_–C_10_ aromatics (2000), C_10_–C_16_ aromatics (1000), and C_16_–C_35_ aromatics (1000)
*PAHs*	GC–MS	SPIMFAB	acenaphthene (Acen, 80), acenaphthylene (Acyl, 80), anthracene (A, 80), benzo[*a*]anthracene (BaA, 80), benzo[*a*]pyrene (BaP, 80), benzo[*b*]fluoranthene (BbF, 80), benzo[*g*,*h*,*i*]perylene (BPer, 80), benzo[*k*]fluoranthene (BkF, 80), chrysene (Chry, 80), dibenzo[*a*,*h*]anthracene (DahA, 80), fluoranthene (Fluo, 80), fluorene (F, 80), indeno[1,2,3-*cd*]pyrene (IP, 80), naphthalene (Nap, 80), phenanthrene (Phen, 80), and pyrene (Pyr, 80)
*BTEX / MTBE; chlorobenzenes; chlorinated aliphatics*	GC−MS, GC−ECD (tetra−hexa chlorobenzenes)	US EPA 8260, US EPA 5021A, US EPA 5021, MADEP 2004, rev. 1.1 and ISO 15009, US EPA 8081 (tetra−hexa chlorobenzenes)	benzene (20), ethylbenzene (20), methyl tert-butyl ether (MTBE, 50), styrene (40), toluene (100), and sum of xylenes (15); monochlorobenzene (10), 1,2-dichlorobenzene (20), 1,3-dichlorobenzene (20), 1,4-dichlorobenzene (20), 1,2,3-trichlorobenzene (20), 1,2,4-trichlorobenzene (30), 1,3,5- trichlorobenzene (50), 1,2,3,4-tetrachlorobenzene (10), 1,2,3,5 + 1,2,4,5-tetrachlorobenzene (20), pentachlorobenzene (10), hexachlorobenzene (HclB, 5), diclobenil (10), and quintozene−pentachloroaniline sum (20); dichloromethane (800), 1,1-dichloroethane (10), 1,2-dichloroethane (100), 1,2-dichloropropane (100), trichloromethane (chloroform, 30), tetrachloromethane (10), hexachloroethane (10), 1,1-dichloroethene (10), cis-1,2-dichloroethene (20), trans-1,2-dichloroethene (10), 1,1,1-trichloroethane (10), 1,1,2-trichloroethane (40), trichloroethene (10), tetrachloroethene (20), and vinyl chloride (100)
*chlorophenols*	GC–MS/GC-ECD	US EPA 8041, US EPA 3500, and DIN ISO 14154	2-monochlorophenol (20), 3-monochlorophenol (20), 4-monochlorophenol (20), 2,3-dichlorophenol (20), 2,4 + 2,5-dichlorophenol (40), 2,6-dichlorophenol (20), 3,4-dichlorophenol (20), 3,5-dichlorophenol (20), 2,3,4-trichlorophenol (20), 2,3,5-trichlorophenol (20), 2,3,6-trichlorophenol (20), 2,4,5-trichlorophenol (20), 2,4,6-trichlorophenol (20), 3,4,5-trichlorophenol (20), 2,3,5,6-tetrachlorophenol (20), 2,3,4,5-tetrachlorophenol (20), 2,3,4,6-tetrachlorophenol (20), and pentachlorophenol (PClPh, 20)
*aldehydes*	HPLC		formaldehyde (100), acetaldehyde (200), propional (200), butanal (200), and glutaraldehyde (pentanedial, 200)
*PCBs*	GC–MS	DIN ISO 10382	2,4,4′-trichlorobiphenyl (PCB 28, 0.1), 2,2′,5,5′-tetrachlorobiphenyl (PCB 52, 0.1), 2,2′,4,5,5′-pentachlorobiphenyl (PCB 101, 0.1), 2,3′,4,4′,5′-pentachlorobiphenyl (PCB 118, 0.1), 2,2′,3,4,4′,5′-hexachlorobiphenyl (PCB 138, 0.1), 2,2′,4,4′,5,5′-hexachlorobiphenyl (PCB 153, 0.1), and 2,2′,3,4,4′,5,5′-heptachlorobiphenyl (PCB 180, 0.1)
*alkylphenols*^a^	GC–MS		4-*tert*-octylphenol (OP, 10–130) and 4-nonylphenol (NP, 100–200)
*phthalates*^a^	GC–MS	standard: DIN 19742	dimethyl phthalate (DMP, 50), diethyl phthalate (DEP, 50), di-*n*-propyl phthalate (DPP, 50–120), di-*n*-butyl phthalate (DBP, 50–80), diisobutyl phthalate (DiBP, 50–110), di-*n*-pentyl phthalate (DNPP, 50), di-*n*-octyl phthalate (DNOP, 50–7000), di-2-ethylhexyl phthalate (DEHP, 50), butylbenzylphthalate (BBP, 50–200), dicyclohexyl phthalate (DCP, 50–60), diisodecyl phthalate (DIDP, 10000), diisononyl phthalate (DINP, 2500–100,000), and di-*n*-hexylphthalate (DNHP, 50–100)
*brominated flame retardants*^a^	LC–MS/MS	DIN 38414	2,4,4′-tribromodiphenyl ether (BDE 28, 0.032–0.48), 2,2′,4,4′-tetrabromodiphenyl ether (BDE 47, 0.16–0.5), 2,2′,4,4′,5-pentabromodiphenyl ether (BDE 99, 0.18–0.5), 2,2′,4,4′,6-penta-bromodiphenyl ether (BDE 100, 0.064–0.48), 2,2′,4,4′,5,5′-hexabromodiphenyl ether (BDE 153, 0.13–0.48), 2,2′,4,4′,5,6′-hexabromodiphenyl ether (BDE 154, 0.027–0.48), tetrabromobisphenol A (TBBP-A, 5), decabromobiphenyl (DeBB, 9–21), and hexabromocyclododecane (HBCD, 50)
*PFASs*	LC–MS/MS		perfluoro-*n*-butanoic acid (PFBA, 0.5), perfluoro-*n*-pentanoic acid (PFPeA, 0.5), perfluoro-*n*-hexanoic acid (PFHxA, 0.5), perfluoro-*n*-heptanoic acid (PFHpA, 0.5), perfluoro-*n*-octanoic acid (PFOA, 0.5), perfluoro-*n*-nonanoic acid (PFNA, 0.5), perfluoro-*n*-decanoic acid (PFDA, 0.5), PFUnDA perfluoro-*n*-undecanoic acid (PFUnDA, 0.5), perfluoro-*n*-dodecanoic acid (PFDoDA, 0.5), perfluorobutanesulfonic acid (PFBS, 0.5), perfluorohexanesulfonic acid (PFHxS, 0.5), perfluoroheptanesulfonic acid (PFHpS, 0.5), perfluorooctanesulfonic acid (PFOS, 0.5), perfluorodecanesulfonic acid (PFDS, 0.5), perfluorooctanesulfonamide (FOSA, 0.5), 6:2 fluorotelomer sulfonate (6:2 FTS, 0.5), 8:2 fluorotelomer sulfonate (8:2 FTS, 0.5), perfluoro-*n*-tridecanoic acid (PFTrDA, 0.5), perfluoro-*n*-tetradecanoic acid (PFTeDA, 0.5), *N*-methyl perfluorooctane sulfonamide (MeFOSA, 0.5), *N*-ethyl perfluorooctane sulfonamide (EtFOSA, 0.5), *N*-methyl perfluorooctane sulfonamidoethanol (MeFOSE, 0.5), and *N*-ethyl perfluorooctane sulfonamidoethanol (EtFOSE, 0.5)
*organotins*	GC–MS	ISO 23161:2011	monobutyltin (MBT, 1), dibutyltin (DBT, 1), tributyltin (TBT, 1), tetrabutyltin (TetBT, 1), monooctyltin (MOT, 1), dioctyltin (DOT, 1), tricyclohexyltin (TCHT, 1), monophenyltin (MPhT, 1), diphenyltin (DPhT, 1), and triphenyltin (TPhT, 1)
*pesticides*	GC-ECD (organochlorine pesticides) and LC–MS/MS (other pesticides)	US EPA 8081 (organochlorine pesticides) and CSN EN 15637 (other pesticides)	acetamiprid (10), acetochlor (10), alachlor (10), aldicarb (10), aldicarb sulfone (10), aldicarb sulfoxide (10), aldrin (10), ametryn (10), atrazine (10), atrazine-desisopropyl (10), azoxystrobin (10), boscalid (10), cadusafos (10), carbaryl (10), carbendazim (10), carbofuran (10), carbofuran-3-hydroxy (10), chlorfenvinphos (10), chloridazon (10), chloridazon-desphenyl (10), chloridazon-methyldesphenyl (10), 6-chloronicotinic acid (10), chlorpyrifos (10), chlorsulfuron (10), chlortoluron (10), clomazone (10), clothianidin (10), cyanazine (10), cyproconazole (10), atrazine-desethyl (10), terbuthylazine-desethyl (10), atrazine-desisopropyl (10), o,p′-DDD (10), p,p′-DDD (10), o,p′-DDE (10), p,p′-DDE (10), o,p′-DDT (10), p,p′-DDT (10), desmetryn (10), diazinon (10), difenacoum (10), diflufenican (10), dichlorvos (10), dicrotophos (10), dieldrin (10), dimethoate (10), dimoxystrobin (10), diuron (10), endrin (10), alpha-endosulfan (10), epoxiconazole (10), fenoxycarb (10), fipronil (10), fipronil sulfone (10), fluazifop (10), fonofos (10), phorate (10), phosalone (10), phosphamidon (10), phosmet (10), phosmet oxon (10), heptachlor (10), *cis*-heptachlor epoxide (10), *trans*-heptachlor epoxide (10), hexazinone (10), alpha-HCH (10), beta-HCH (10), gamma-HCH (lindane, 10), 2-hydroxyatrazine (10), hydroxy-terbutylazine (10), imidacloprid (10), imidacloprid olefin (10), imidacloprid urea (10), indoxacarb (10), isodrin (10), isoproturon (10), isoproturon-desmethyl (10), isoproturon-monodesmethyl (10), kresoxim-methyl (10), linuron (10), malaoxon (10), malathion (10), metamitron (10), metazachlor (10), methidathion (10), methiocarb (10), methiocarb sulfone (10), methiocarb sulfoxide (10), metconazole (10), metolachlor (isomers) (10), methomyl (10), methomyl oxime (10), metribuzin (10), oxamyl (10), pendimethalin (10), pethoxamid (10), pirimicarb (10), prochloraz (10), prometon (10), prometryn (10), propazine (10), propiconazole (10), propoxur (10), pyrimethanil (10), sebuthylazine (10), simazine (10), simazine-2-hydroxy (10), simetryn (10), tebuconazole (10), telodrin (10), terbuthylazine-desethyl-2-hydroxy (10), terbutryn (10), terbuthylazine (10), thiacloprid (10), and thiamethoxam (10)

a The LOQs varied between samples due to matrix effects, so their ranges are presented.

### Data Analysis

Because at least one sample had a concentration below the LOQ for all substances, much of the data generated by this study is left-censored (i.e., only an upper limit for a given concentration is known). When analyzing such data, statistical methods were employed for the analysis of censored data.^52^ The significance of correlations was tested using the nonparametric Kendall’s tau test, and significance of differences between groups was tested using the Peto & Peto generalized Wilcoxon test, both implemented with the Nondetects and Data Analysis for Environmental Data package (NADA) in R. Statistical analysis was only applied to substances quantified in at least 25% of samples. Correlations between noncensored data were tested using the Spearman’s rank-order correlation test, while significant differences were tested using the Wilcoxon test, both nonparametric.

Factors of variation within or between ponds were calculated as the ratio of the highest to lowest concentration, setting concentrations below the LOQ equal to the LOQ, making these factors of variation equal to the lower limit of actual variability.

## Results and Discussion

### Substance Occurrence

**Figure 1:**
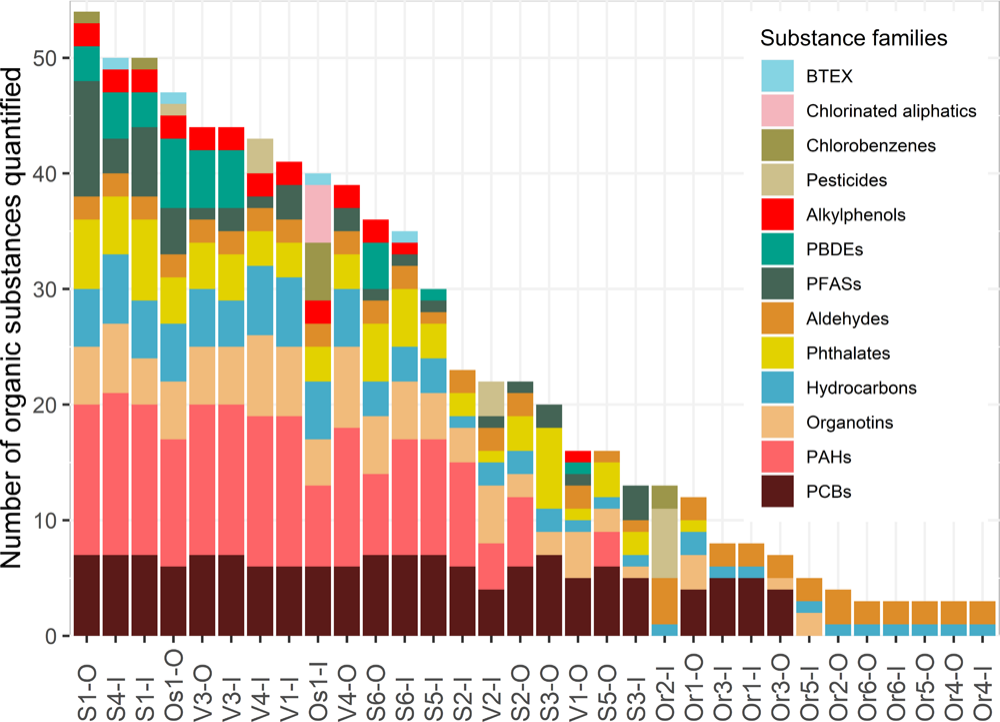
Total number of quantified organic substances per sample (*n*_quant_) according to the substance family. Sample names refer to samples taken from ponds in Stockholm (S), Östersund (Os), Växjö(V), and Örebro (Or) at inlet(I) and outlet (O).

Among the 259 substances analyzed, 92 were quantified in at least one sample (see Table S2 for the list of all substances according to the frequency of quantification (*f*_quant_) and Table S3 for *f*_quant_ of each quantified substance). The most recurrent substance families were hydrocarbons and aldehydes (Figure 1). PAHs, PCBs, phthalates, and organotins were all quantified in majority of samples, while PFASs, PBDEs, and alkylphenols were quantified in over 25% of samples. Other substance families including BTEX, chlorinated aliphatics, chlorobenzenes, and pesticides were rarely quantified (<13% of samples). A total of 167 substances were never quantified, including a majority of pesticides and chlorinated organics.

The number of substances quantified in a given sample (*n*_quant_) varied from 3 to 52 (Figure 1). Several substance families were much more frequently quantified in the 20 samples from the cities of Östersund, Stockholm, and Växjö (OSV) than in the 12 samples from Örebro. Reasons for these differences will be discussed further in a subsequent section.

Among the six samples with the highest *n*_quant_, all OSV samples, a common contamination profile emerged, which includes PCBs, PAHs, organotins, hydrocarbons, phthalates, aldehydes, PBDEs, PFASs, and alkylphenols. The rarely quantified substance families (pesticides, chlorobenzenes, chlorinated aliphatic, and BTEX) occurred sporadically in different samples.

Significant correlations (Kendall’s tau test P < 0.01, see Table S4 for P and tau values) were observed between *n*_quant_ and concentrations of individual substances including C_10_–C_12_, C_12_–C_16_, and C_16_–C_35_ aliphatic hydrocarbons, C_16_–C_35_ aromatic hydrocarbons, 10 PAHs (Phen, Fluo, Pyr, BaA, Chry, BbF, BkF, BaP, BPer, and IP), two aldehydes (formaldehyde and acetaldehyde), all seven PCBs, a PBDE (BDE 99), a PFAS (PFOS), both alkylphenols (OP and NP), four phthalates (DBP, DEHP, DiDP, and DiNP), and five organotins (MBT, DBT,TBT, MOT, and DOT). This list includes substances from all families found to be recurrent in the most contaminated samples and supports the hypothesis of the existence of a typical urban contamination profile (i.e., a group of substances tending to occur together in similar ratios), the strength of which depends on various site-specific factors, including substance and particle sources. It also indicates that *n*_quant_ is a good indicator of overall contamination for this data set as it corresponds to both the complexity and magnitude of contamination.

### Concentrations of Organic Substances

**Figure 2:**
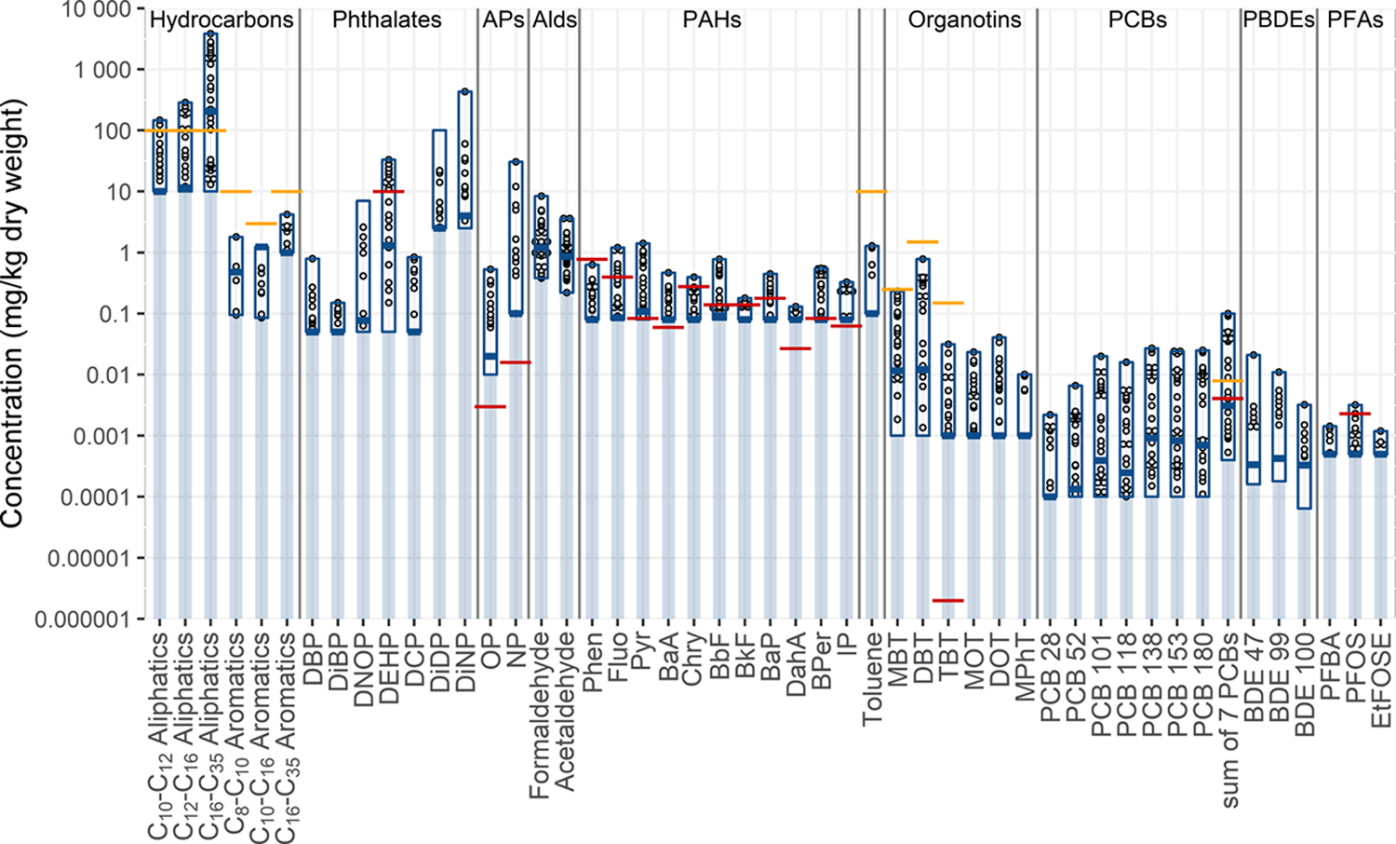
Minimum, median, and maximum concentrations (dark blue boxes) of substances quantified in at least 10% of stormwater sediment samples (*n* = 32) compared with Swedish guidelines for sensitive land use of contaminated sites^57^ shown in yellow and Norwegian environmental quality standards for sediments^58^ shown in red. The dark blue boxes were constructed by replacing values below the LOQ with the LOQ; as no substances were quantified in all samples, the true distributions extend below this box to an unknown extent as represented by the pale blue line. Quantified concentrations are shown by black circles.

Figure 2 shows the observed concentrations of substances quantified in more than 10% of samples. The following section presents the results for key congeners from each family. All results are summarized numerically in Table S3 of the Supporting Information.

#### Hydrocarbons and PAHs

Both aliphatic hydrocarbons and PAHs are hydrophobic substances;^53^ thus, their accumulation in sediments is expected to be a major fate process.

Among hydrocarbons, C_16_–C_35_ aliphatics were quantified most frequently (97%) and at the highest concentrations (<10–3820 mg/kg). Aromatic hydrocarbons occurred less frequently and at concentrations several orders of magnitude below those of aliphatic hydrocarbons. Within each family, heavy species (C_16_–C_35_) were found at higher concentrations than lighter-weight species. These results, both in terms of observed concentrations and relative abundance of species, are similar to those reported for sediments from 13 stormwater ponds^31^ and a sedimentation facility^49^ treating road runoff in the Gothenburg region of Sweden.

Overall, PAHs occurred in 53% of samples, with Σ_16_PAH concentrations between 0.2 and 6.4 mg/kg (median 0.64 mg/kg), in the lower range of those previously reported in the literature. Both a study of 16 stormwater ponds in South Carolina, USA^54^ and gully pot sediments from Drammen and Oslo, Norway reported PAH concentrations in the range of those observed in this study. However, previous studies of PAHs in stormwater sediments in Minnesota, USA^34^ and Ontario, Canada,^38,39^ the particulate phase of stormwater from various sites in the Paris region of France,^15,16,55^ and gully pot sediments from Bergen, Norway^56^ all reported markedly higher PAH concentrations than those observed in this study.

When PAHs were quantified in a sample, Pyr was always present usually (in 70% of cases) at the highest concentration of any PAH molecule. Fluo, BbF, and Phen also occurred frequently (in 50, 50, and 38% of all samples) and occasionally had the highest concentration of any PAH in a sample (in 18, 6, and 6% of cases).

Overall, heavy PAHs (4–6 molecular rings) dominated over light-weight PAHs (2–3 rings), indicating that combustion processes rather than fossil fuel leaks are the main sources of PAHs in the studied catchments.^59^ It should be noted that coal tar, which is known to be a major source of PAHs in the urban environment in the United States,^34,60^ has not been used in Swedish road construction since 1973.^61^

#### Phthalates

The hydrophobicity of phthalates varies greatly with the molecular weight, and heavier phthalates thus have a greater propensity to accumulate in sediments. For example, log K_OW_ for DMP is in the range of 1.5–1.9 while that for DEHP is in the range of 3.6–9.7.^53^

DEHP was the most frequently quantified phthalate (66% of samples) with concentrations ranging from <0.05 to 33 mg/kg (median 1.3 mg/kg), a variability of nearly three orders of magnitude. DiNP reached higher concentrations than DEHP (up to 430 mg/kg) but was less frequently quantified (31% of samples), most likely due to its higher LOQ. DBP and DiDP were also quantified in over a quarter of samples (31 and 28%, respectively), reaching concentrations of up to 0.79 and 22 mg/kg.

Most previous studies analyzing phthalates in stormwater sediments and particles have detected them,^16,44,62,63^ with the exception of a study of stormwater pond sediments in Florida in which DBP was never detected due to a very high LOQ (a 100-fold higher than in the present study).^35^ DEHP concentrations measured in sediments from 15 stormwater ponds in Minnesota^44^ and a sedimentation tank treating highway runoff in Gothenburg, Sweden^62^ were of the same order as those in the present study, with the Gothenburg samples corresponding to the most contaminated samples in this study. However, phthalate concentrations measured in the particulate phase of stormwater in the Paris region (DEHP from a dense urban catchment^16^ and DEHP, DBP, and DiBP concentrations from a heavily trafficked road^63^) were distinctly higher than those in this study.

#### Alkylphenols

Both NP and OP, relatively hydrophobic molecules likely to accumulate in sediments,^64^ were quantified in 38% of samples, most often occurring together. A high intersite variability was observed, and concentrations of NP (<0.1 to 30.5 mg/kg) were typically higher than those of OP (<0.02 to 0.53 mg/kg), as would be expected due to the fact that NPs account for a higher proportion of industrial applications of alkylphenols than OPs.^65^

NP concentrations previously observed in sediments from a detention basin in an industrial area of Lyon, France^42^ and a stormwater tank in Gothenburg, Sweden,^43^ as well as in the particulate phase of stormwater from a dense urban catchment^16^ and a heavily trafficked road^63^ in the Paris region of France all fit within the range observed in this study. These same studies observed OP either in the same range as the present study^16,42^ or at higher concentrations.^49,63^ Interestingly, high concentrations of OP appear to be associated with catchments containing heavily trafficked roads, both in the scientific literature^49,63^ and this study; this may be due to the presence of OP in tires,^66^ at least in the European market. By contrast, a study of sediments from 15 stormwater ponds in Minnesota observed NP more frequently (100% of samples) and at higher concentrations than the present study but never detected OP, despite lower LOQs than the present study.^44^

#### Aldehydes

Formaldehyde and acetaldehyde concentrations ranged from <0.38 to 8.4 mg/kg and < 0.22 to 3.6 mg/kg, respectively, in the present study, on the same order as those measured in previous Swedish studies of sediments from a stormwater sedimentation facility^49^ and in fine particles from street-sweeping dust^67^ (1.2–5.7 mg/kg and 1.1–7.6 mg/kg for formaldehyde and acetaldehyde, respectively). Although there are no regulatory limits for aldehydes in sediments, 22% of samples exceeded the probable no-effect concentrations (PNEC) in freshwater sediments for formaldehyde of 2.3 mg/kg;^68^ no sediment PNEC could be found for acetaldehyde.

Aldehydes are products of incomplete combustion known to be present in vehicular exhaust and may also be formed due to secondary reactions of hydrocarbons in the atmosphere.^69^ As their physical–chemical properties (high volatility, low hydrophobicity, and high degradability) are not expected to favor their persistence and accumulation in sediments,^70,71^ their prevalence in stormwater pond sediments in this study is somewhat surprising. One explanation is that very high gaseous concentrations in vehicle exhaust result in non-negligible concentrations in emitted particles (probably accounting for a small proportion of emitted mass). As fugacity modeling has shown that both formaldehyde and acetaldehyde tend to remain in the medium to which they are emitted,^70,71^ it is possible that these compounds persist once such particles settle in stormwater ponds. An alternative hypothesis is that aldehydes are secondary contaminants formed due to reactions of other components of the sediment. Indeed, a previous study has shown that acetaldehyde can be formed in natural sediments through fermentation of organic matter under anoxic conditions;^72^ however, as the reported concentrations were several orders of magnitude lower than those observed in this study for similar organic carbon concentrations, this hypothesis alone cannot explain the levels observed.

#### Organotins

Organotins are relatively hydrophobic and tend to partition to the solid phase in sediments.^73^ DBT was the most recurrent organotin, present in 69% of samples with a median concentration of 12 mg/kg (range < 1 to 781 mg/kg), while MBT was close behind, quantified in 66% of samples with a median concentration of 12 mg/kg (range < 1 to 231 mg/kg). TBT was quantified less frequently (44% of samples) and at lower concentrations (up to 31.3 mg/kg).

Organotins, which are used in PVC, antifouling paints, and timber preservatives,^74^ have previously been quantified in urban stormwater sediments collected from manholes and pumping stations in Oslo and Drammen, Norway^75^ as well as in the particulate phase of stormwater in a dense urban catchment in the Paris region.^16^ Both studies found TBT at much higher concentrations (up to 11,000 mg/kg^75^ and 200 mg/kg^16^), representing a higher proportion of overall organotins than the present study. Concentrations of MBT and DBT also tended to be higher than those in the present study, though to a lesser extent.

#### PCBs

Despite the prohibition of PCBs in Sweden in 1972 and a concerted effort to remove existing PCBs from Swedish buildings since 1998,^76^ PCBs were some of the most frequently detected substances in this study likely due to the fact that the removal of PCB-containing materials is a long, arduous process, which is as yet incomplete.^76^ Indeed, of the seven analyzed PCB species, five PCBs (101, 118, 138, 153, and 180) were quantified in 69–75% of samples, while PCB 28 and PCB 53 were quantified in 28 and 53% of samples, respectively. As highly hydrophobic substances,^53^ PCBs are expected to accumulate in sediments.

PCB concentrations generally followed the order 138 > 153 > 180 > 101 > 118 > 52 > 28, mirroring that observed by Zgheib et al.^16^ in the particulate phase of stormwater from a dense urban catchment in the Paris region, with the exception of PCB 28, which was observed at concentrations between those of PCB 180 and PCB 101. The median Σ_7_PCB concentration was 3.2 μg/kg (range < 0.4 to 100 μg/kg), while median concentrations of each congener ranged from <0.1 to 0.94 μg/kg (overall range < 0.1 to 27 μg/kg). While these concentrations were lower than those observed by Zgheib et al. (congener median < 10–50 μg/kg and overall range 10–60 μg/kg)^16^ and a study of gully pot sediments in Bergen, Norway (Σ_7_PCB median 29 μg/kg and range < 0.4 to 704 μg/kg),^56^ they tended to be higher than those observed in a recent study of PCBs in the particulate phase of stormwater in Maryland, USA (overall congener range < 0.00167 to 1.92 μg/kg).^77^

#### PBDEs

The most frequently quantified brominated flame retardant was a PBDE, BDE 99 (*f*_quant_ = 25%), with concentrations ranging from 0.18 to 11 μg/kg. Both BDE 47 and BDE 100 were quantified in 22% of samples with concentrations between <0.16 and 21 μg/kg and < 0.064 μg/kg, respectively. PBDEs are very hydrophobic,^53^ so their accumulation in sediments is expected. Several previous studies have analyzed and either very rarely (3% of samples)^41^ or never^16,42^ quantified PBDEs in stormwater sediments^41,42^ or in the particulate phase of stormwater,^16^ which may be explained at least in part by higher LOQs than those of the present study. PBDEs have also been quantified in 100% of sediment samples from 15 stormwater ponds in Minnesota with concentrations very similar to those in this study.

#### PFASs

Although the accumulation in sediments is not expected to be a major fate process of PFASs due to their relatively low partition coefficients (e.g., log *K*_OC_ = 2.68 for PFOS),^78^ over 50% of samples contained at least one PFAS. The most recurrent PFAS was PFOS (*f*_quant_ = 44%), with concentrations ranging from <0.5 to 3.18 μg/kg. PFASs were also analyzed in sediments from 15 stormwater ponds in Minnesota, where PFOS was also the most frequently detected species, quantified in 80% of samples at similar concentrations (<0.33 to 2.25 μg/kg). PFOA, never detected in the present study, was quantified in 40% of samples from the Minnesota study, generally at concentrations below our LOQ.^44^ Fire-fighting foams are thought to be the main source of PFASs, though they have also been found in a variety of products including food, personal care products, ski wax, clothing, paper, and paints.^79^ Among these, paints, in particular, are likely to be a diffuse source of PFASs in the urban environment. Although they were not detected at all sites, the recurrence of PFASs in the present study adds to a growing body of evidence that urban runoff is chronically contaminated by diffuse sources in the urban environment and may be an important vector of PFASs.^80−82^

#### Pesticides

Among the pesticides analyzed in this study, a great majority (101 of 114) were never quantified and none were quantified in more than two samples (*f*_quant_ = 6%, see Table S3). The highest concentrations were observed for DDT and its degradation products (concentrations up to 1.58 mg/kg for p,p′-DDT), while quantified concentrations of contemporary pesticides (terbuthylazine-desethyl-2-hydroxy, hydroxyl-terbuthylazine, carbendazim, propiconazole, and terbutryn) were between 0.01 and 0.021 mg/kg.

Although pesticides have recently been shown to be the most prevalent organic substances in urban stormwater by a vast screening study across 21 sites across the USA,^8^ many modern pesticides are relatively hydrophilic^53^ and tend to be in the dissolved rather than particulate phase in stormwater.^16^ As such, they are unsusceptible to sedimentation as a treatment process^83^ and unexpected to accumulate in sediments, which is likely the main reason for the relative rarity of pesticides in the present study. Contemporary pesticides are often relatively biodegradable,^53^ which may also limit their accumulation in sediments. Another contributing factor may be differences in pesticide use between countries, for example, in 2012, around 26,000 metric tons of pesticides were sold for household use in the USA^84^ (0.032 metric tons/km^2^ urban land area^85^) vs 674 metric tons in Sweden^86^ (0.022 tons/km^2^). Sweden also has very strict regulations as to the use of pesticides in urban amenity areas,^87^ which likely limits sources from both public and private areas.

In previous studies of stormwater pond sediments, DDT and its degradation products have occasionally been detected,^35,41^ as in the present study, as well as a number of pesticides that were analyzed but never detected in this study, including chlorpyrifos,^41,42^ dichlorvos,^41^ fonofos,^41^ endosufan,^35,41^ endrin,^35^ dieldrin,^35^ diuron,^42^ and isoproturon.^42^ Again, these differences may, at least in part, be due to differences in pesticide regulation and use between countries.

### Variability of Contamination

The following section presents an analysis of the factors influencing the extent of contamination by focusing on inter- and intrasite variability of *n*_quant_ and the 34 substances quantified in at least 25% of samples (subst >25%).

#### Intersite Variability

The sediment quality varied greatly between sites. Even considering the limiting hypothesis that nonquantified samples had concentrations equal to the LOQ (which underestimates variability), C_16_–C_35_ aliphatic hydrocarbons, PCBs 101, 118, 156, 138, and 180, NP, DEHP, DiNP, MBT, and DBT all had factors of variation exceeding 100 (see Table S5). Aldehydes and PAHs showed less variability (factors of variation 16–22 and 4–18, respectively) despite frequent quantification, indicating that sources of these substances may be less site-specific.

No significant differences were observed for *n*_quant_ between land-use type (Wilcoxon P > 0.01). However, concentrations of several substances were significantly lower in residential catchments than in industrial/commercial and road catchments (P < 0.01, Table S6), including hydrocarbons (C_10_–C_12_, C_12_–C_16_, and C_16_–C_35_ aliphatics), PAHs (Phen, Fluo, Pyr, and BPer), PCBs (PCB 52 and 118), an alkylphenol (OP), phthalates (DEHP, DiDP, and DiNP), and organotins (MBT, DBT, MOT, and DOT).

No significant correlations were observed between *n*_quant_ and site properties including catchment area, facility-to-catchment area ratio, facility age, sediment age (either the age of the facility or the time since sediment was last emptied), and catchment imperviousness (Spearman P > 0.01) nor between these properties and most substance concentrations (Kendall P > 0.01, Table S7). The three exceptions to this were significant positive correlations between sediment age and concentrations of BaP, IP, and acetaldehyde, though the tau values were low (0.285, 0.275, and 0.414, respectively).

As previously mentioned, many substances were much more frequently quantified in samples from OSV than in samples from Örebro. This was the case for PCBs (quantified in 100% of OSV samples vs 33% of Örebro samples), PAHs (85% vs 0%), organotins (100% vs 33%), phthalates (100% vs 8%), PFASs (85% vs 0%), PBDEs (45% vs 0%), and alkylphenols (65% vs 0%). A significant difference was observed in *n*_quant_ between Örebro and Stockholm (P = 0.00034) and Örebro and Växjö (P = 0.0020), though not between Örebro and Östersund (P = 0.12) probably due to the fact than only one pond was sampled in Östersund. Significant differences were also observed between concentrations in Örebro and Växjö for 32/34 subst >25%, between Örebro and Stockholm for 28/34, and between Örebro and Östersund for 23/34 (P < 0.01, see Table S8). These differences cannot be explained by land use alone as four of the Örebro catchments were industrial/commercial, while two were residential.

Field observations indicate that the sediments are likely composed of different types of particles. Sediments collected from the ponds in Örebro tended to be fine, sticky, dense, and gray in color (see Figure S1a), whereas sediments from OSV (particularly those from sites with industrial/commercial catchments) were usually black, looser, and less adherent (Figure S1b). Among the remaining OSV sites, three were residential catchments, while one was a highway catchment. Sediments from one of the residential catchments resembled peat (Figure S1c), while sediments from the other three sites were brown and sandy (Figure S1d).

Interestingly, the loose, black sediments account for the 12 most contaminated sediments. Given these observations, we hypothesize that the loose, black sediments are primarily composed of anthropogenic particles (e.g., wear particles and soot), which carry the urban signature. This hypothesis builds on previous studies, which have observed black, anthropogenic particles in stormwater pond sediments using a microscope coupled to micro X-ray fluorescence (μXRF).^88^

At each site, the anthropogenic particles may be diluted, to a greater or lesser extent, by other, less (or differently) contaminated sources of particles, including eroded soil, sand, and natural organic matter. Soil may be eroded, for example, from permeable surfaces within a catchment or from open channels carrying stormwater to the pond, which we hypothesize is the case in Örebro, where four of the facilities (Or-2, Or-4, Or-5, and Or-6) received water through open channels and the other two catchments (Or-1 and Or-3) had relatively low imperviousness (22 and 40%). This hypothesis is supported by lower C/N ratios in sediments from Örebro than in those from other cities (statistically significant with respect to sediments from both Stockholm and Växjö, Wilcoxon P < 0.01).

To explore this hypothesis, future research should focus on developing and using methods for identifying sources of particles within a sediment, which can then be used to normalize substance concentrations to compare signatures of anthropogenic particles from different catchment types and locations. As the signatures of these particles are likely to be more stable than those of the resulting sediment, quantifying anthropogenic particles in a sediment could be a surrogate for expensive analysis of the wide variety of substances that may be present in stormwater sediments.

#### Intrasite Variability

The *n*_quant_ measured in inlet samples correlated significantly with the *n*_quant_ measured in outlet samples from the same facilities (Spearman P = 3.8E-6, rho = 0.9); correlations were also observed between inlet and outlet concentrations for 22 of the 34 subst >25% (Kendall’s tau test P < 0.01, Table S9). These correlations are expected since the quality of the sediment at each point is supposed to be influenced by the quality of runoff from the catchment.

However, high variability was observed between the contamination of inlet and outlet samples from a given pond. The median factor of variation for *n*_quant_ (ratio of highest to lowest number of quantified substances within a pond) between two samples within the same pond was 1.18; for one pond (Or-2), it reached 3.25 (13 substances detected at the inlet vs 4 at the outlet). Variation was even higher for individual substance concentrations. Among subst >25%, the median factor of variation (ratio of highest to lowest concentration in a pond) ranged from 1.0 to 1.8; maximum factors of variation among these substances ranged from 3.1 to 60 (see Table S10). This high degree of variability is even more remarkable given that each sample was a composite of at least four cores and underlines the sensitivity of conclusions about a pond’s level of contamination to sampling strategies.

Previous studies have found sediments near pond inlets to be less contaminated than sediments farther downstream in the pond, which has been attributed to the slower settling time of smaller particles, which tend to have higher concentrations of organic substances.^27,41,89^ However, in this study, the differences in inlet and outlet concentrations do not appear to be systematic. No significant differences were observed between *n*_quant_ for inlet and outlet samples (paired Wilcoxon P = 0.55) or between inlet and outlet concentrations of individual substances (paired Peto–Peto Wilcoxon P in the range of 0.289–0.990, see Table S11).

### Environmental Implications

#### Disposal of Stormwater Sediments

Results from this study emphasize the importance of considering hydrophobic organic contaminants during environmental risk assessment of stormwater sediments. Indeed, observed concentrations of at least one substance exceeded the Swedish contaminated site guidelines for sensitive land use (G-SLU)^57^ for 22 of the 32 samples (see Figure S2a), including all OSV samples and two Örebro samples. This implies that upland disposal options will be limited due to a potential risk to terrestrial ecosystems and/or human health.^90^ The most critical contaminants with respect to the G-SLU, both in terms of frequency and magnitude of exceedance (see Figure S3a) were C_15_–C_35_ aliphatic hydrocarbons followed by high-molecular-weight PAHs and Σ_7_PCBs. It should be noted that the G-SLU only applies to substances typically associated with contaminated sites (chlorinated organics, Σ_7_PCBs, PAHs, BTEX, MTBE, and aromatic and aliphatic hydrocarbons).^57^

The high variability of contamination between ponds shows that the risk associated with stormwater pond sediments differs greatly between sites; sediment management strategies should therefore be adapted to the level of risk posed by each site. The least contaminated sediments in this study likely do not require a specific treatment of organic substances prior to upland disposal due to the dilution of pollution sources by a large proportion of natural particles. However, a higher proportion of natural particles also means that the mass of the sediment generated per mass of the pollutant retained is higher, entailing a more frequent need for heavy maintenance activities (i.e., sediment removal); as such, facilities should not intentionally be designed to accumulate natural particles.

Variations in the sediment quality within ponds underline the importance of establishing a representative sampling strategy when evaluating environmental risk, while the absence of systematic variations in contamination between inlet and outlet samples does not support differing sediment management depending on the location within a pond.

#### Conflicts between Water Quality Improvement and Habitat Functions of Stormwater Ponds

This work shows that the retention of organic contaminants in stormwater ponds for their water quality improvement function may compromise their function as a habitat for aquatic life.

To demonstrate this, observed concentrations were compared to Norwegian environmental quality standards for sediments (EQS-S),^58^ which are designed to protect 100% of aquatic species, assuming equilibrium partitioning between sediments and water,^90^ and applied to 28 of the European Union priority substances, including Σ_7_PCBs, several PAH molecules, and pesticides, as well as two alkylphenols (NP and OP), a phthalate (DEHP), two PFASs (PFOA and PFOS), and two organotins (TBT and TPhT). Indeed, 22 of the 32 sediment samples had quantified concentrations exceeding the EQS-S for at least one substance (Figure S2b), most frequently PAHs (Pyr, BPer, BaA, BbF, and IP), Σ_7_PCBs, TBT, OP, NP, and DEHP. The greatest magnitudes of exceedance were observed for TBT followed by NP, p,p′-DDT (which was, however, only quantified in two samples), OP, Σ_7_PCBs, Pyr, and HClB (see Figures S3b,c).

The existence of a tension between the water quality and habitat functions of stormwater ponds should not deter the implementation of stormwater ponds; indeed, in the absence of a treatment facility, the contamination would be shifted to natural water bodies, where it would also compromise the ecosystem’s health. However, it does imply that stakeholders must make a value judgment as to the relative importance of the water quality and habitat functions of stormwater ponds.^91^ Where ecosystem protection is a priority, chemical analysis may be complemented with bioassays and ecological surveys to fully characterize the ecological risks of the complex contaminant mixture in stormwater pond sediments.^18,92,93^

#### Design Considerations for Stormwater Infrastructures

From an engineering perspective, including a sedimentation forebay upstream of a stormwater pond may offer some protection to its ecosystem by limiting the pollutant load reaching the pond, while reducing the required frequency of sediment removal.

It should also be noted that by their nature, wet stormwater ponds, which maintain a permanent pool of water, do not provide favorable conditions for the biodegradation of pollutants after their retention. Other types of green infrastructures, such as stormwater biofilters, which are designed to achieve aerobic conditions between storm events, may be more effective in dissipating retained pollutants.^94,95^

## Supporting Information Available

Facility details, additional sample results, results of statistical analysis, photographs of sediment, and a comparison of concentrations with quality standards (PDF).

## Abbreviations

*BaA*: benzo[*a*]anthracene
*BaP*: benzo[*a*]pyrene
*BbF*: benzo[*b*]fluoranthene
*BDE*: bromodiphenylether
*BkF*: benzo[*k*]fluoranthene
*BPer*: benzo[*g*,*h*,*i*]perylene
*BTEX*: benzene, toluene, ethylbenzene, and xylenes
*Chry*: chrysene
DahA: dibenzo[*a*,*h*]anthracene
*DBP*: di-*n*-butylphthalate
*DBT*: dibutyltin
*DCP*: dicyclohexylphthalate
*DEHP*: di-2-ethylhexylphthalate
*DiBP*: diisobutylphthalate
*DiDP*: diisodecylphthalate
*DiNP*: diisononylphthalate
*DNOP*: di-*n*-octylphthalate
*DOT*: dioctyltin
*ECD*: electron capture detector
*EQS-S*: environmental quality standards for sediment
*EtFOSE*: *N*-ethylperfluorooctanesulfonamidoethanol
*f*_quant_: frequency of quantification
*Fluo*: fluoranthene
GC: gas chromatography
*G-SLU*: guidelines for sensitive land use
*HPLC*: high-performance liquid chromatography
*IP*: indeno[1,2,3-*cd*]pyrene
*LC*: liquid chromatography
*LOQ*: limit of quantification
*MBT*: monobutyltin
*MOT*: monooctyltin
*MPhT*: monophenyltin
*MS*: mass spectrometry
*MTBE*: methyl *tert*-butyl ether
*NP*: 4-nonylphenol
*n*_quant_: number of substances quantified
*OP*: 4-*tert*-octylphenol
*OSV*: Östersund, Stockholm, and Växjö
*PAH*: polycyclic aromatic hydrocarbon
*PBDE*: polybrominated diphenyl ether
*PCB*: polychlorinated biphenyl
*PFAS*: perfluorinated substances
*PFBA*: perfluoro-*n*-butanoic acid
*PFOA*: perfluoro-*n*-octanoic acid
*PFOS*: perfluorooctanesulfonic acid
*Phen*: phenanthrene
*PNEC*: probable no-effect concentration
*Pyr*: pyrene
*SCM*: stormwater control measure
*TBT*: tributyltin
*TPhT*: triphenyltin
*US EPA*: United States Environmental Protection Agency.
