# Supplementary material for: Contamination of Urban Stormwater Pond Sediments: A Study of 259 Legacy and Contemporary Organic Substances
Source: Environ Sci Technol. 2021 Feb 19;55(5):3009–20. doi: 10.1021/acs.est.0c07782 (PMC8026099; doi:10.1021/acs.est.0c07782)
Supplement: Supplementary file 1 — es0c07782_si_001.pdf [file es0c07782_si_001.pdf]

**Supporting information for:**

# Contamination of Urban Stormwater Pond Sediments: A Study of 259 Legacy and Contemporary Organic Substances

*Kelsey Flanagan,[1]* Godecke-Tobias Blecken,[1] Heléne Österlund,[1] Kerstin Nordqvist,[1] Maria Viklander[1]*

[1]Urban Water Engineering, Department of Civil, Environmental and Natural Resources Engineering, Luleå University of Technology, 971 87 Luleå, Sweden

***Corresponding author:**

**kelsey.flanagan@ltu.se**

26 pages; 11 tables; 3 figures

## I. Characteristics of studied facilities

| Facility | Municipality | Facility type | Catchment classification | Catchment imperviousness | Catchment surface area (ha) | Type of inlet | Year of construction | Facility volume (m3) | Facility surface area (ha) |
|---|---|---|---|---|---|---|---|---|---|
| *Ös-1* | Östersund | Pond | Industrial / commericial | **97** | **91** | **Pipe** | **2000** | **9100** | **0.45** |
| *Ör-1* | Örebro | Pond | Industrial / commericial | **40** | **40** | **Pipe** | **1996** | **1300** | **0.13** |
| *Ör-2* | Örebro | Pond | Industrial / commericial | **50** | **57** | **Open channel** | **2000** | **ND** | **0.48** |
| *Ör-3* | Örebro | Pond | Residential | **22** | **1490** | **Pipe** | **2010** | **20500** | **1.78** |
| *Ör-4* | Örebro | Pond | Residential | **5** | **14.5** | **Open channel** | **2007** | **ND** | **0.33** |
| *Ör-5* | Örebro | Pond | Industrial / commericial | **80** | **30** | **Open channel** | **2006** | **5800** | **0.5** |
| *Ör-6* | Örebro | Pond | Industrial / commericial | **75** | **31** | **Open channel** | **2006** | **5800** | **0.8** |
| *S-1* | Stockholm | Pond | Industrial / commericial | **ND** | **78** | **Pipe** | **2009** | **1450** | **0.17** |
| *S-2* | Stockholm | Pond | Residential | **ND** | **304** | **Pipe** | **2007** | **530** | **0.05** |
| *S-3* | Stockholm | Pond | Residential | **ND** | **770** | **Pipe** | **1997** | **ND** | **0.06** |
| *S-4* | Stockholm | Pond | Road / highway | **50** | **2** | **Pipe** | **2002** | **498** | **0.03** |
| *S-5* | Stockholm | Pond | Road / highway | **ND** | **ND** | **ND** | **ND** | **ND** | **0.06** |
| *S-6* | Stockholm | Subsurface sedimentation tank | Road / highway | **100** | **1.14** | **Pipe** | **1997** | **132** | **0.006** |
| *V-1* | Växjö | Pond | Industrial / commericial | **46** | **519** | **Pipe / open channel** | **1989** | **3300** | **0.36** |
| *V-2* | Växjö | Pond | Residential | **30** | **69** | **Pipe / open channel** | **2008** | **6600** | **0.36** |
| *V-3* | Växjö | Pond | Industrial / commericial | **60** | **330** | **Pipe / open channel** | **1994** | **17600** | **1.1** |
| *V-4* | Växjö | Pond | Industrial / commericial | **54** | **7.5** | **Pipe** | **1988** | **870** | **0.06** |

**Table S1.** Characteristics of studied facilities. ND means that data was unavailable for a given site.

## II. Frequency of quantification of all substances

| Frequency | Substances | | | |
|---|---|---|---|---|
| **Never quantified** | $C_5$-$C_8$ Aliphatics | Styrene | Dimoxystrobin | Methiocarb |
| | $C_8$-$C_{10}$ Aliphatics | MTBE | Diuron | sulfoxide |
| | Nap | Aldrin | Epoxiconazole | Metconazole |
| | Acyl | Dieldrin | Fenoxycarb | Metolachlor |
| | Acen | Endrin | Fipronil | Methomyl |
| | A | Isodrin | Fipronil sulfone | Methomyl |
| | Dichloromethane | Telodrin | Fluazifop | oxime |
| | 1,1-dichloroethane | alpha-HCH | Fonofos | Metribuzin |
| | 1,2-dichloroethane | beta-HCH | Phorate | Oxamyl |
| | 1,2-dichloropropane | Lindane | Phosalone | Pendimethalin |
| | Chloroform | Heptachlor | Phosphamidon | Pethoxamid |
| | Tetrachloromethane | cis-Heptachlor | Phosmet | Pirimicarb |
| | Trans-1,2-dichloroethene | epoxide | Phosmet oxon | Prochloraz |
| | 1,1,1-trichloroethane | trans-Heptachlor | Hexazinone | Prometon |
| | Trichloroethene | epoxide | 2-hydroxyatrazine | Prometryn |
| | Vinyl chloride | alpha-Endosulfan | Imidacloprid | Propazine |
| | Monochlorobenzene | Simazine-2-hydroxy | Imidacloprid olefin | Propoxur |
| | 1,2,3-trichlorobenzene | Carbofuran-3- | Imidacloprid urea | Pyrimethanil |
| | 1,2,3,4-tetrachlorobenzene | hydroxy | Indoxacarb | Sebuthylazine |
| | 1,2,3,5+1,2,4,5- | 6-chloronicotinic | Isoproturon | Simazine |
| | tetrachlorobenzene | acid | Isoproturon-desmethyl | Simetryn |
| | Diclobenil | Acetamiprid | Isoproturon- | Tebuconazole |
| | Quintozene- | Acetochlor | monodesmethyl | Terbuthylazine |
| | pentachloroaniline | Alachlor | Cadusafos | Thiacloprid |
| | 2-monochlorophenol | Aldicarb | Carbaryl | Thiamethoxam |
| | 3-monochlorophenol | Aldicarb sulfone | Carbofuran | Pentanedial |
| | 4-monochlorophenol | Aldicarb sulfoxide | Clomazone | DeBB |
| | 2,3-dichlorophenol | Ametryn | Chlorfenvinphos | HBCD |
| | 2,4 + 2,5-dichlorophenol | Atrazine | Chloridazon | PFHpA |
| | 2,6-dichlorophenol | Azoxystrobin | Chloridazon-desphenyl | PFOA |
| | 3,4-dichlorophenol | Boscalid | Chloridazon- | PFNA |
| | 3,5-dichlorophenol | Cyanazine | methyldesphenyl | PFDA |
| | 2,3,4-trichlorophenol | Cyproconazole | Chlorpyrifos | PFBS |
| | 2,3,5-trichlorophenol | Atrazine-desethyl | Chlorsulfuron | PFHxS |
| | 2,3,6-trichlorophenol | Terbuthylazine- | Chlortoluron | PFHpS |
| | 2,4,5-trichlorophenol | desethyl | Clothiadinin | 6:2 FTS |
| | 2,4,6-trichlorophenol | Atrazine- | Kresoxim-methyl | 8:2 FTS |
| | 3,4,5-trichlorophenol | desisopropyl | Linuron | PFTrDA |
| | 2,3,5,6-tetrachlorophenol | Desmetryn | Malaoxon | DEP |
| | 2,3,4,5-tetrachlorophenol | Diazinon | Malathion | DNPP |
| | 2,3,4,6-tetrachlorophenol | Difenacoum | Metamitron | DNHP |
| | Pentachlorophenol | Diflufenican | Metazachlor | TetBT |
| | Benzene | Dichlorvos | Methidathion | TCHT |
| | Ethylbenzene | Dicrotophos | Methiocarb | TPhT |
| | Xylene | Dimethoate | Methiocarb sulfone | |
| **$0<f_{quant}\le25\%$** | $C_8$-$C_{10}$ Aromatics | Hexachlorobenzene | Propional | FOSA |
| | $C_{16}$-$C_{35}$ Aromatics | Toluene | Butanal | PFTeDA |
| | F | o,p'-DDT | BDE 28 | MeFOSA |
| | BkF | p,p'-DDT | BDE 47 | EtFOSA |
| | DahA | o,p'-DDD | BDE 99 | MeFOSE |
| | Hexachloroethane | p,p'-DDD | BDE 100 | EtFOSE |
| | Cis-1,2-dichloroethene | o,p'-DDE | BDE 153 | DMP |
| | 1,1,2-trichloroethane | p,p'-DDE | BDE 154 | DPP |
| | Tetrachloroethene | Terbuthylazine- | TBBP-A | DBP |
| | 1,1-dichloroethene | desethyl-2- | PFBA | DiBP |
| | 1,2-dichlorobenzene | hydroxy | PFPeA | DNOP |
| | 1,3-dichlorobenzene | Hydroxy- | PFHxA | BBP |
| | 1,4-dichlorobenzene | terbutylazine | PFUnDA | DCP |
| | 1,2,4-trichlorobenzene | Carbendazim | PFDoDA | MPhT |
| | 1,3,5-trichlorobenzene | Propiconazole | PFDS | DPhT |
| | Pentachlorobenzene | Terbutryn | | |

| Frequency | Substances | | | |
|---|---|---|---|---|
| $25<f_{quant}\leq50\%$ | $C_{10}$-$C_{12}$ Aliphatics<br>$C_{10}$-$C_{16}$ Aromatics<br>Phen<br>Fluo<br>BaA | Chry<br>BbF<br>BaP<br>BPer<br>IP | PCB 28<br>PFOS<br>OP<br>NP<br>DBP | DiDP<br>DiNP<br>TBT<br>MOT<br>DOT |
| $50<f_{quant}\leq75\%$ | $C_{12}$-$C_{16}$ Aliphatics<br>Pyr<br>PCB 52 | PCB 101<br>PCB 118<br>PCB 153 | PCB 138<br>PCB 180<br>DEHP | MBT<br>DBT |
| $75<f_{quant}<100\%$ | $C_{16}$-$C_{35}$ Aliphatics | Formaldehyde | Acetaldehyde | |

**Table S2: List of substances by frequency of quantification in stormwater pond sediments, n=32**

## III. Summary of quantified substances

| Substance group | Substance | $f_{quant}$ (%) | Concentrations | | |
|---|---|---|---|---|---|
| | | | Median | Minimum | Maximum |
| Hydrocarbons | $C_{10}$-$C_{12}$ Aliphatics (mg/kg TS) | 41% | <10 | <10 | 146 |
| | $C_{12}$-$C_{16}$ Aliphatics (mg/kg TS) | 53% | 11.5 | <10 | 285 |
| | $C_5$-$C_{16}$ Aliphatics (mg/kg TS) | 53% | 18 | <11 | 410 |
| | $C_{16}$-$C_{35}$ Aliphatics (mg/kg TS) | 97% | 205 | <10 | 3820 |
| | $C_8$-$C_{10}$ Aromatics (mg/kg TS) | 16% | <0.48 | <0.095 | 1.8 |
| | $C_{10}$-$C_{16}$ Aromatics (mg/kg TS) | 28% | <1.24 | <0.085 | 1.24 |
| | $C_{16}$-$C_{35}$ Aromatics (mg/kg TS) | 25% | <1 | <1 | 4.2 |
| Polycyclic aromatic hydrocarbons (PAHs) | F (mg/kg TS) | 6% | <0.08 | <0.08 | 0.095 |
| | Phen (mg/kg TS) | 38% | <0.08 | <0.08 | 0.628 |
| | Fluo (mg/kg TS) | 50% | 0.0855 | <0.08 | 1.2 |
| | Pyr (mg/kg TS) | 53% | 0.109 | <0.08 | 1.41 |
| | BaA (mg/kg TS) | 38% | <0.08 | <0.08 | 0.464 |
| | Chry (mg/kg TS) | 41% | <0.08 | <0.08 | 0.395 |
| | BbF (mg/kg TS) | 50% | 0.0935 | <0.08 | 0.773 |
| | BkF (mg/kg TS) | 25% | <0.08 | <0.08 | 0.179 |
| | BaP (mg/kg TS) | 34% | <0.08 | <0.08 | 0.444 |
| | DahA (mg/kg TS) | 22% | <0.08 | <0.08 | 0.13 |
| | BPer (mg/kg TS) | 44% | <0.08 | <0.08 | 0.548 |
| | IP (mg/kg TS) | 31% | <0.08 | <0.08 | 0.328 |
| | Sum of 16 PAH (mg/kg TS) | 53% | 0.64 | <0.2 | 6.4 |
| | Carcinogenic PAH (mg/kg TS) | 50% | 0.28 | <0.11 | 2.6 |
| | Sum other PAH (mg/kg TS) | 53% | 0.36 | <0.092 | 3.8 |
| | Sum medium-weight PAH (mg/kg TS) | 53% | 0.215 | <0.092 | 3.2 |
| | Sum high-weight PAH (mg/kg TS) | 50% | 0.32 | <0.11 | 3.2 |
| BTEX | Toluene (mg/kg TS) | 13% | <0.1 | <0.1 | 1.28 |
| Chlorinated aliphatics | Hexachloroethane (mg/kg TS) | 3% | <0.01 | <0.01 | 0.126 |
| | Cis-1,2-dichloroethene (mg/kg TS) | 3% | <0.04 | <0.04 | 0.18 |
| | 1,1,2-trichloroethane (mg/kg TS) | 3% | <0.01 | <0.01 | 0.125 |
| | Tetrachloroethene (mg/kg TS) | 3% | <0.02 | <0.02 | 0.094 |
| | 1,1-dichloroethene (mg/kg TS) | 3% | <0.01 | <0.01 | 0.167 |
| Chlorobenzenes | 1,2-dichlorobenzene (mg/kg TS) | 3% | <0.02 | <0.02 | 0.69 |
| | 1,3-dichlorobenzene (mg/kg TS) | 3% | <0.02 | <0.02 | 0.22 |
| | 1,4-dichlorobenzene (mg/kg TS) | 3% | <0.02 | <0.02 | 0.47 |
| | 1,2,4-trichlorobenzene (mg/kg TS) | 3% | <0.03 | <0.03 | 0.31 |
| | 1,3,5-trichlorobenzene (mg/kg TS) | 3% | <0.05 | <0.05 | 0.39 |
| | Pentachlorobenzene (mg/kg TS) | 3% | <0.01 | <0.01 | 0.02 |
| | sum tetra- and pentachlorobenzenes (mg/kg TS) | 3% | <0.02 | <0.017 | 0.04 |
| | Hexachlorobenzene (mg/kg TS) | 9% | <0.005 | <0.005 | 0.168 |
| Aldehydes | Formaldehyde (mg/kg TS) | 97% | 1.2 | <0.38 | 8.4 |
| | Acetaldehyde (mg/kg TS) | 88% | 0.86 | <0.22 | 3.6 |
| | Propional (mg/kg TS) | 3% | <0.2 | <0.2 | 0.5 |
| | Butanal (mg/kg TS) | 6% | <0.2 | <0.2 | 0.5 |

| Substance group | Substance | $f_{quant}$ (%) | Concentrations | | |
|---|---|---|---|---|---|
| | | | Median | Minimum | Maximum |
| Polychlorinated Biphenyls (PCBs) | PCB 28 (µg/kg TS) | 28% | <0.1 | <0.1 | 2.2 |
| | PCB 52 (µg/kg TS) | 53% | 0.135 | <0.1 | 6.6 |
| | PCB 101 (µg/kg TS) | 75% | 0.415 | <0.1 | 20 |
| | PCB 118 (µg/kg TS) | 69% | 0.26 | <0.1 | 16 |
| | PCB 153 (µg/kg TS) | 75% | 0.865 | <0.1 | 24 |
| | PCB 138 (µg/kg TS) | 75% | 0.935 | <0.1 | 27 |
| | PCB 180 (µg/kg TS) | 72% | 0.7 | <0.1 | 25 |
| | sum of 7 PCBs (µg/kg TS) | 75% | 3.2 | <0.4 | 100 |
| Alkylphenols | OP (mg/kg TS) | 38% | <0.02 | <0.01 | 0.529 |
| | NP (mg/kg TS) | 38% | <0.1 | <0.1 | 30.5 |
| Phthalates | DMP (mg/kg TS) | 3% | <0.05 | <0.05 | 0.099 |
| | DPP (mg/kg TS) | 6% | <0.05 | <0.05 | 0.7 |
| | DiBP (mg/kg TS) | 19% | <0.05 | <0.05 | 0.15 |
| | DBP (mg/kg TS) | 31% | <0.05 | <0.05 | 0.79 |
| | DNOP (mg/kg TS) | 22% | <0.0775 | <0.05 | 7 |
| | DEHP (mg/kg TS) | 66% | 1.3 | <0.05 | 33 |
| | BBP (mg/kg TS) | 6% | <0.05 | <0.05 | 0.2 |
| | DCP (mg/kg TS) | 22% | <0.05 | <0.05 | 0.84 |
| | DiDP (mg/kg TS) | 28% | <2.5 | <2.5 | 22 |
| | DiNP (mg/kg TS) | 31% | <4 | <2.5 | 430 |
| Brominated flame retardants | BDE 28 (µg/kg TS) | 3% | <0.26 | <0.032 | 0.7 |
| | tetraBDE (µg/kg TS) | 9% | <3.3 | <2 | 21 |
| | BDE 47 (µg/kg TS) | 22% | <0.335 | <0.16 | 21 |
| | pentaBDE (µg/kg TS) | 9% | <4 | <2.4 | 15 |
| | BDE 99 (µg/kg TS) | 25% | <0.425 | <0.18 | 11 |
| | BDE 100 (µg/kg TS) | 22% | <0.33 | <0.064 | 3.2 |
| | BDE 153 (µg/kg TS) | 9% | <0.275 | <0.13 | 1.4 |
| | BDE 154 (µg/kg TS) | 9% | <0.235 | <0.027 | 0.6 |
| | decaBDE (µg/kg TS) | 3% | <25.5 | <10 | 110 |
| | TBBP-A (µg/kg TS) | 9% | <5 | <5 | 6.66 |
| Perfluorinated substances (PFASs) | PFBA (µg/kg TS) | 19% | <0.5 | <0.5 | 1.42 |
| | PFPeA (µg/kg TS) | 6% | <0.5 | <0.5 | 0.895 |
| | PFHxA (µg/kg TS) | 3% | <0.5 | <0.5 | 0.635 |
| | PFUnDA (µg/kg TS) | 3% | <0.5 | <0.5 | 0.872 |
| | PFDoDA (µg/kg TS) | 6% | <0.5 | <0.5 | 1.46 |
| | PFOS (µg/kg TS) | 44% | <0.5 | <0.5 | 3.18 |
| | PFDS (µg/kg TS) | 9% | <0.5 | <0.5 | 0.557 |
| | FOSA (µg/kg TS) | 9% | <0.5 | <0.5 | 0.994 |
| | PFTeDA (µg/kg TS) | 6% | <0.5 | <0.5 | 1.35 |
| | MeFOSA (µg/kg TS) | 3% | <0.5 | <0.5 | 0.584 |
| | EtFOSA (µg/kg TS) | 9% | <0.5 | <0.5 | 0.735 |
| | MeFOSE (µg/kg TS) | 3% | <0.5 | <0.5 | 0.825 |
| | EtFOSE (µg/kg TS) | 13% | <0.5 | <0.5 | 1.19 |

| Substance group | Substance | $f_{quant}$ (%) | Concentrations | | |
|---|---|---|---|---|---|
| | | | Median | Minimum | Maximum |
| Organotins | MBT (µg/kg TS) | 66% | 12 | <1 | 231 |
| | DBT (µg/kg TS) | 69% | 12 | <1 | 781 |
| | TBT (µg/kg TS) | 44% | <1 | <1 | 31.3 |
| | MOT (µg/kg TS) | 47% | <1 | <1 | 23.4 |
| | DOT (µg/kg TS) | 47% | <1 | <1 | 40.7 |
| | MPhT (µg/kg TS) | 13% | <1 | <1 | 10.1 |
| | DPhT (µg/kg TS) | 6% | <1 | <1 | 2.69 |
| Pesticides | o,p'-DDT (mg/kg TS) | 3% | <0.01 | <0.01 | 0.25 |
| | p,p'-DDT (mg/kg TS) | 6% | <0.01 | <0.01 | 1.58 |
| | o,p'-DDD (mg/kg TS) | 3% | <0.01 | <0.01 | 0.328 |
| | p,p'-DDD (mg/kg TS) | 3% | <0.01 | <0.01 | 0.869 |
| | o,p'-DDE (mg/kg TS) | 3% | <0.01 | <0.01 | 0.02 |
| | p,p'-DDE (mg/kg TS) | 3% | <0.01 | <0.01 | 0.216 |
| | Terbuthylazine-desethyl-2-hydroxy (mg/kg TS) | 3% | <0.01 | <0.01 | 0.1 |
| | Hydroxy-terbutylazine (mg/kg TS) | 3% | <0.01 | <0.01 | 0.1 |
| | Carbendazim (mg/kg TS) | 3% | <0.01 | <0.01 | 0.1 |
| | Propiconazole (mg/kg TS) | 6% | <0.01 | <0.01 | 0.1 |
| | Terbutryn (mg/kg TS) | 3% | <0.01 | <0.01 | 0.1 |

**Table S3: Frequency of quantification ($f_{quant}$), minimal, maximal and median concentrations for compounds quantified in at least one sample**

## IV. Correlation test results between quantified concentrations of each substance and the total number of substances quantified in each sample

| Substance | $f_{censored}$ | P-value | tau |
|---|---|---|---|
| $C_{10}$-$C_{12}$ Aliphatics* | 0.59 | 4.25E-07 | 0.542 |
| $C_{12}$-$C_{16}$ Aliphatics* | 0.47 | 2.07E-08 | 0.649 |
| $C_{16}$-$C_{35}$ Aliphatics* | 0.03 | 5.16E-09 | 0.726 |
| $C_{10}$-$C_{16}$ Aromatics | 0.72 | 0.963 | 0.006 |
| $C_{16}$-$C_{35}$ Aromatics* | 0.75 | 2.42E-05 | 0.373 |
| Phen* | 0.63 | 6.18E-07 | 0.520 |
| Fluo* | 0.50 | 1.01E-08 | 0.653 |
| Pyr* | 0.47 | 2.6E-09 | 0.690 |
| BaA* | 0.63 | 2.22E-07 | 0.540 |
| Chry* | 0.59 | 2.86E-07 | 0.550 |
| BbF* | 0.50 | 1.25E-08 | 0.649 |
| BkF* | 0.75 | 4.02E-05 | 0.363 |
| BaP* | 0.66 | 4.01E-07 | 0.512 |
| BPer* | 0.56 | 2.63E-08 | 0.611 |
| IP* | 0.69 | 5.76E-06 | 0.442 |
| Formaldehyde* | 0.03 | 0.00175 | 0.389 |
| Acetaldehyde* | 0.13 | 0.00398 | 0.357 |
| BDE 99* | 0.75 | 0.00278 | 0.359 |
| PCB28* | 0.72 | 0.00062 | 0.369 |
| PCB52* | 0.47 | 3.18E-09 | 0.685 |
| PCB101* | 0.25 | 5.37E-10 | 0.762 |
| PCB118* | 0.31 | 7.17E-09 | 0.704 |
| PCB153* | 0.25 | 7E-11 | 0.800 |
| PCB138* | 0.25 | 5.62E-11 | 0.804 |
| PCB180* | 0.28 | 9.11E-11 | 0.792 |
| sum of 7 PCBs* | 0.25 | 4.55E-11 | 0.808 |
| PFOS* | 0.56 | 5.9E-05 | 0.442 |
| OP* | 0.63 | 1.49E-05 | 0.500 |
| NP* | 0.63 | 3.87E-07 | 0.542 |
| DBP* | 0.69 | 0.00017 | 0.389 |
| DEHP* | 0.34 | 4.19E-09 | 0.710 |
| DiDP* | 0.72 | 0.00681 | 0.300 |
| DiNP* | 0.69 | 0.00406 | 0.329 |
| Monobutyltin* | 0.34 | 7.67E-09 | 0.698 |
| Dibutyltin* | 0.31 | 2.16E-09 | 0.728 |
| Tributyltin* | 0.56 | 0.000127 | 0.421 |
| Monooctyltin* | 0.53 | 2.94E-06 | 0.524 |
| Dioctyltin* | 0.53 | 1.23E-07 | 0.593 |

**Table S4:** Results of the Kendall's tau test between censored substance concentrations**,** n=32**.** Significant correlations, considering $\alpha$=0.01 are marked with a * The test was only computed for substances quantified in at least 8 of the 32 analyzed samples ($f_{cens} \leq 75\%$ censored).

## V. Statistics addressing inter-site variability

| Substance | $f_{censored}$ | Inter-site factor of variation |
|---|---|---|
| $C_{10}$-$C_{12}$ Aliphatics | 0.60 | 14.6 |
| $C_{12}$-$C_{16}$ Aliphatics | 0.50 | 28.5 |
| $C_{16}$-$C_{35}$ Aliphatics | 0.03 | 382 |
| $C_{10}$-$C_{16}$ Aromatics | 0.73 | 14.6 |
| Phen | 0.63 | 7.9 |
| Fluo | 0.50 | 15.0 |
| Pyr | 0.50 | 17.6 |
| BaA | 0.63 | 5.8 |
| Chry | 0.60 | 4.9 |
| BbF | 0.53 | 5.6 |
| BaP | 0.67 | 5.6 |
| BPer | 0.57 | 6.9 |
| IP | 0.70 | 4.1 |
| Formaldehyde | 0.03 | 22.1 |
| Acetaldehyde | 0.13 | 16.4 |
| PCB28 | 0.73 | 22.0 |
| PCB52 | 0.47 | 66.0 |
| PCB101 | 0.27 | 200 |
| PCB118 | 0.30 | 160 |
| PCB153 | 0.27 | 240 |
| PCB138 | 0.27 | 270 |
| PCB180 | 0.30 | 250 |
| PFOS | 0.60 | 6.4 |
| OP | 0.63 | 52.9 |
| NP | 0.63 | 305 |
| DBP | 0.70 | 15.8 |
| DEHP | 0.37 | 660 |
| DiDP | 0.70 | 40.0 |
| DiNP | 0.67 | 172 |
| Monobutyltin | 0.37 | 231 |
| Dibutyltin | 0.33 | 781 |
| Tributyltin | 0.60 | 31.3 |
| Monooctyltin | 0.57 | 23.4 |
| Dioctyltin | 0.57 | 40.7 |

**Table S5:** Summary of factors of variability within ponds for substances quantified in at least 25% of samples

| Substance | $f_{censored}$ | P-value (all) | P-value (IC-Res) | P-value (IC-Ro) | P-value (Res-Ro) |
|---|---|---|---|---|---|
| $C_{10}$-$C_{12}$ Aliphatics | 0.60 | 0.030 | **0.0013** | 0.91 | **0.0013** |
| $C_{12}$-$C_{16}$ Aliphatics | 0.50 | 0.12 | **0.0071** | 0.83 | **0.0071** |
| $C_{16}$-$C_{35}$ Aliphatics | 0.03 | 0.042 | **0.00080** | 0.60 | **0.00082** |
| $C_{10}$-$C_{16}$ Aromatics | 0.73 | 0.018 | 1 | **0.0047** | 1 |
| Phen | 0.63 | 0.011 | **0.0013** | 0.18 | **0.0013** |
| Fluo | 0.50 | 0.041 | **0.00082** | 0.38 | **0.00082** |
| Pyr | 0.50 | 0.041 | **0.00032** | 0.35 | **0.00032** |
| BaA | 0.63 | 0.14 | 0.045 | 0.75 | 0.045 |
| Chry | 0.60 | 0.22 | 0.054 | 0.69 | 0.054 |
| BbF | 0.53 | 0.15 | 0.013 | 0.59 | 0.013 |
| BaP | 0.67 | 0.12 | 0.58 | 0.46 | 0.578515 |
| BPer | 0.57 | 0.049 | **0.0026** | 0.67 | **0.0026** |
| IP | 0.70 | 0.082 | 0.046 | 0.75 | 0.046 |
| Formaldehyde | 0.03 | 0.42 | 0.19 | 0.32 | 0.19 |
| Acetaldehyde | 0.13 | 0.48 | 0.80 | 0.48 | 0.80 |
| PCB28 | 0.73 | 0.048 | 0.011 | 0.10 | 0.011 |
| PCB52 | 0.47 | 0.054 | **0.0071** | 0.19 | **0.0071** |
| PCB101 | 0.27 | 0.15 | 0.012 | 0.31 | 0.012 |
| PCB118 | 0.30 | 0.12 | **0.0038** | 0.31 | **0.0038** |
| PCB153 | 0.27 | 0.21 | 0.012 | 0.52 | 0.012 |
| PCB138 | 0.27 | 0.29 | 0.026 | 0.63 | 0.026 |
| PCB180 | 0.30 | 0.25 | 0.033 | 0.60 | 0.033 |
| PFOS | 0.60 | 0.48 | 0.19 | 0.63 | 0.19 |
| OP | 0.63 | **0.0022** | **0.0094** | 0.04 | **0.0094** |
| NP | 0.63 | 0.027 | 0.046 | 0.39 | 0.046 |
| DBP | 0.70 | 0.27 | 0.12 | 0.22 | 0.12 |
| DEHP | 0.37 | 0.11 | **0.0069** | 0.35 | **0.0069** |
| DiDP | 0.70 | 0.37 | **0.0027** | 0.76 | **0.0027** |
| DiNP | 0.67 | 0.037 | **0.0076** | 0.12 | **0.0076** |
| Monobutyltin | 0.37 | 0.013 | **0.0018** | 0.10 | **0.0018** |
| Dibutyltin | 0.33 | 0.081 | **0.0035** | 0.36 | **0.0035** |
| Tributyltin | 0.60 | 0.18 | 0.054 | 0.63 | 0.053592 |
| Monooctyltin | 0.57 | 0.039 | **0.0045** | 0.24 | **0.0045** |
| Dioctyltin | 0.57 | 0.040 | **0.0026** | 0.76 | **0.0026** |

**Table S6:** Results of the Peto&Peto generalized Wilcoxon test for differences between substance concentrations observed in each city, n=32, $n_{IC}$=18, $n_{Res}$=9, $n_{Ro}$=5. P-values for significant differences, considering α=0.01, are in bold. The tests were only computed for substances for which $f_{censored}$<75%.

| Substance | $f_{censored}$ | P-value (Catchment area) | P-value (Ratio facility to catchment) | P-value (Facility age) | P-value (Sediment age) | P-value (Imperviousness) |
|---|---|---|---|---|---|---|
| $C_{10}$-$C_{12}$ Aliphatics | 0.60 | 0.54 | 0.44 | 0.068 | 0.068 | 0.077 |
| $C_{12}$-$C_{16}$ Aliphatics | 0.50 | 0.70 | 0.36 | 0.11 | 0.25 | 0.16 |
| $C_{16}$-$C_{35}$ Aliphatics | 0.03 | 1 | 0.41 | 0.011 | 0.044 | 0.073 |
| $C_{10}$-$C_{16}$ Aromatics | 0.73 | 0.81 | 0.73 | 0.84 | 0.84 | 0.86 |
| Phen | 0.63 | 0.22 | 0.85 | 0.13 | 0.22 | 0.19 |
| Fluo | 0.50 | 0.62 | 0.41 | 0.18 | 0.097 | 0.14 |
| Pyr | 0.50 | 0.64 | 0.46 | 0.24 | 0.085 | 0.23 |
| BaA | 0.63 | 0.73 | 0.49 | 0.21 | 0.063 | 0.25 |
| Chry | 0.60 | 0.93 | 0.34 | 0.31 | 0.071 | 0.22 |
| BbF | 0.53 | 0.83 | 0.40 | 0.22 | 0.057 | 0.20 |
| BaP | 0.67 | 0.81 | 0.38 | 0.27 | **0.0078** | 0.11 |
| BPer | 0.57 | 0.58 | 0.61 | 0.11 | 0.043 | 0.11 |
| IP | 0.70 | 0.94 | 0.59 | 0.096 | **0.0053** | 0.28 |
| Formaldehyde | 0.03 | 0.86 | 0.50 | 0.042 | 0.021 | 0.35 |
| Acetaldehyde | 0.13 | 0.76 | 0.79 | 0.081 | **0.0013** | 0.25 |
| PCB28 | 0.73 | 0.84 | 0.51 | 0.65 | 0.53 | 0.59 |
| PCB52 | 0.47 | 0.71 | 0.25 | 0.19 | 0.32 | 0.034 |
| PCB101 | 0.27 | 0.51 | 0.05 | 0.058 | 0.58 | 0.33 |
| PCB118 | 0.30 | 0.66 | 0.14 | 0.077 | 0.32 | 0.39 |
| PCB153 | 0.27 | 0.51 | 0.05 | 0.045 | 0.49 | 0.25 |
| PCB138 | 0.27 | 0.36 | 0.03 | 0.063 | 0.54 | 0.28 |
| PCB180 | 0.30 | 0.36 | 0.04 | 0.044 | 0.38 | 0.21 |
| PFOS | 0.60 | 0.66 | 0.10 | 0.36 | 0.81 | 0.44 |
| OP | 0.63 | 0.18 | 0.98 | 0.22 | 0.25 | 0.077 |
| NP | 0.63 | 1 | 0.41 | 0.078 | 0.012 | 0.087 |
| DBP | 0.70 | 0.87 | 0.29 | 0.50 | 0.80 | 0.57 |
| DEHP | 0.37 | 0.87 | 0.07 | 0.077 | 0.75 | 0.12 |
| DiDP | 0.70 | 0.64 | 0.42 | 0.041 | 0.22 | 0.039 |
| DiNP | 0.67 | 0.84 | 0.61 | 0.10 | 0.12 | 0.045 |
| Monobutyltin | 0.37 | 0.45 | 0.64 | 0.027 | 0.12 | 0.13 |
| Dibutyltin | 0.33 | 0.75 | 0.30 | 0.014 | 0.11 | 0.21 |
| Tributyltin | 0.60 | 0.38 | 1 | 0.034 | 0.22 | 1 |
| Monooctyltin | 0.57 | 0.27 | 0.89 | 0.022 | 0.027 | 0.13 |
| Dioctyltin | 0.57 | 0.50 | 0.87 | 0.12 | 0.072 | 0.064 |

**Table S7: Results of** Kendall's tau test for correlation between censored concentrations and catchment and facility properties, n=30 for all except imperviousness for which n=21 P-values for significant correlations, considering $\alpha$=0.01, are in bold. The test was only computed for substances for which $f_{censored}$<75%.

| **Substance** | **$f_{censored}$** | **P-value (all)** | **P-value (Or-Os)** | **P-value (Or-St)** | **P-value (Or-Va)** | **P-value (Os-St)** | **P-value (Os-Va)** | **P-value (St-Va)** |
|---|---|---|---|---|---|---|---|---|
| $C_{10}$-$C_{12}$ Aliphatics | 0.60 | **0.0027** | **6.3E-05** | **0.0036** | **0.00053** | 0.17 | 0.77 | 0.42 |
| $C_{12}$-$C_{16}$ Aliphatics | 0.50 | **0.00030** | **6.3E-05** | **0.0013** | **0.00021** | 0.092 | 0.35 | 0.31 |
| $C_{16}$-$C_{35}$ Aliphatics | 0.03 | **1.6E-06** | **6.3E-05** | **2.7E-06** | **4.6E-06** | 0.041 | 0.77 | 0.062 |
| $C_{10}$-$C_{16}$ Aromatics | 0.73 | 0.90 | 1 | 1 | 1 | 0.58 | 0.65 | 0.48 |
| Phen | 0.63 | 0.018 | 0.014 | **0.0036** | **0.00053** | 0.77 | 1 | 0.89 |
| Fluo | 0.50 | **0.0014** | **6.3E-05** | **8.6E-05** | **0.00053** | 0.92 | 0.42 | 0.75 |
| Pyr | 0.50 | **0.00056** | **6.3E-05** | **8.6E-05** | **6.3E-05** | 0.69 | 0.42 | 0.68 |
| BaA | 0.63 | **0.0095** | 0.014 | **0.0036** | **0.00053** | 0.77 | 0.28 | 0.54 |
| Chry | 0.60 | **0.0044** | 0.014 | **0.0012** | **0.00053** | 0.46 | 0.28 | 0.48 |
| BbF | 0.53 | **0.00059** | **6.3E-05** | **0.00035** | **6.3E-05** | 0.77 | 0.42 | 0.31 |
| BaP | 0.67 | **0.0083** | **6.3E-05** | 0.025 | **0.00053** | 0.28 | 0.42 | 0.36 |
| BPer | 0.57 | **0.0027** | **6.3E-05** | **0.0012** | **0.00053** | 0.40 | 0.42 | 0.37 |
| IP | 0.70 | **0.0054** | 0.014 | 0.025 | **0.00053** | 0.91 | 0.28 | 0.12 |
| Formaldehyde | 0.03 | **0.00024** | 0.35 | **0.0040** | **4.5E-05** | 0.91 | 1 | 0.011 |
| Acetaldehyde | 0.13 | **0.000018** | 0.063 | 0.24 | **2.3E-05** | 0.46 | 0.88 | **0.00040** |
| PCB28 | 0.73 | 0.014 | 1 | **0.0012** | 0.055 | 0.35 | 0.43 | 0.57 |
| PCB52 | 0.47 | **0.00062** | **6.3E-05** | **1.7E-05** | **0.00053** | 0.85 | 0.89 | 0.79 |
| PCB101 | 0.27 | **0.000087** | **6.3E-05** | **2.7E-06** | **0.00010** | 0.85 | 0.42 | 0.55 |
| PCB118 | 0.30 | **0.00037** | **6.3E-05** | **6.0E-06** | **0.0013** | 0.85 | 0.59 | 0.43 |
| PCB153 | 0.27 | **0.000026** | **6.3E-05** | **3.0E-06** | **8.1E-06** | 0.54 | 0.42 | 0.40 |
| PCB138 | 0.27 | **0.000025** | **6.3E-05** | **4.0E-06** | **5.3E-06** | 0.54 | 0.59 | 0.38 |
| PCB180 | 0.30 | **0.000015** | **6.3E-05** | **2.7E-06** | **4.6E-06** | 0.28 | 0.78 | 0.28 |
| PFOS | 0.60 | **0.0052** | 0.014 | **0.0012** | **6.3E-05** | 1 | 0.88 | 0.82 |
| OP | 0.63 | **0.0099** | **6.3E-05** | **0.0098** | **0.00053** | 0.88 | 0.53 | 0.59 |
| NP | 0.63 | **0.0011** | **6.3E-05** | 0.025 | **6.3E-05** | 0.17 | 0.77 | 0.15 |
| DBP | 0.70 | **0.0093** | 1 | **0.0012** | **0.0082** | 0.19 | 0.27 | 0.77 |
| DEHP | 0.37 | **6.2E-05** | **6.3E-05** | **7.2E-06** | **4.6E-06** | 0.85 | 0.89 | 0.75 |
| DiDP | 0.70 | **0.0023** | **6.3E-05** | **0.0027** | **0.0018** | 0.18 | 0.92 | 0.32 |
| DiNP | 0.67 | **0.0027** | **6.3E-05** | **0.0024** | **0.0031** | 0.18 | 0.81 | 0.44 |
| Monobutyltin | 0.37 | **7.7E-05** | **6.3E-05** | **3.1E-05** | **4.6E-06** | 1 | 0.42 | 0.44 |
| Dibutyltin | 0.33 | **7.2E-06** | **6.3E-05** | **4.5E-06** | **4.6E-06** | 0.85 | 0.42 | 0.093 |
| Tributyltin | 0.60 | **0.0011** | 0.46 | 0.065 | **0.00056** | 0.69 | 0.14 | 0.024 |
| Monooctyltin | 0.57 | **0.00026** | **6.3E-05** | **0.0036** | **4.6E-06** | 0.27 | 0.55 | 0.17 |
| Dioctyltin | 0.57 | **3.3E-11** | **6.3E-05** | **0.0036** | **4.6E-06** | **0.00013** | **0.0023** | 0.49 |

**Table S8:** Results of the Peto&Peto generalized Wilcoxon test for differences between substance concentrations observed in each city, n=32, $n_{Os}$=2, $n_{Or}$=12, $n_{St}$=11, $n_{Va}$=7. P-values for significant differences, considering α=0.01, are in bold. The tests were only computed for substances for which $f_{censored}$<75%.

## VI. Statistics comparing inlet and outlet concentrations

| Substance | $f_{censored}$ | P-value | tau |
|---|---|---|---|
| $C_{10}$-$C_{12}$ Aliphatics* | 0.60 | 0.0052 | 0.381 |
| $C_{12}$-$C_{16}$ Aliphatics* | 0.50 | 0.0097 | 0.419 |
| $C_{16}$-$C_{35}$ Aliphatics* | 0.03 | 0.00063 | 0.667 |
| $C_{10}$-$C_{16}$ Aromatics | 0.73 | 1 | -0.010 |
| Phen | 0.63 | 0.018 | 0.305 |
| Fluo* | 0.50 | 0.00093 | 0.533 |
| Pyr* | 0.50 | 0.00076 | 0.543 |
| BaA | 0.63 | 0.016 | 0.295 |
| Chry | 0.60 | 0.015 | 0.333 |
| BbF | 0.53 | 0.011 | 0.390 |
| BaP* | 0.67 | 0.00023 | 0.419 |
| BPer* | 0.57 | 0.0032 | 0.419 |
| IP | 0.70 | 0.015 | 0.257 |
| Formaldehyde | 0.03 | 0.164 | 0.276 |
| Acetaldehyde | 0.13 | 0.0198 | 0.448 |
| PCB28 | 0.73 | 0.047 | 0.276 |
| PCB52* | 0.47 | 0.0021 | 0.514 |
| PCB101* | 0.27 | 0.00025 | 0.695 |
| PCB118* | 0.30 | 0.00066 | 0.638 |
| PCB153* | 0.27 | 4.6E-05 | 0.771 |
| PCB138* | 0.27 | 5.6E-05 | 0.762 |
| PCB180* | 0.30 | 0.00020 | 0.695 |
| PFOS* | 0.60 | 0.0061 | 0.448 |
| OP* | 0.63 | 0.0014 | 0.438 |
| NP | 0.63 | 0.014 | 0.305 |
| DBP* | 0.70 | 0.00023 | 0.667 |
| DEHP | 0.37 | 0.042 | 0.305 |
| DiDP | 0.70 | 0.043 | 0.314 |
| DiNP* | 0.67 | 0.00035 | 0.648 |
| Monobutyltin* | 0.37 | 0.00055 | 0.638 |
| Dibutyltin* | 0.33 | 0.0065 | 0.371 |
| Tributyltin* | 0.60 | 0.0098 | 0.381 |
| Monooctyltin* | 0.57 | 1.2E-05 | 0.638 |
| Dioctyltin* | 0.57 | 0.0061 | 0.448 |

**Table S9:** Results of the Kendall's tau test for correlation between censored inlet and outlet substance concentrations, n=15 paired inlet-outlet concentrations. Significant correlations considering $\alpha$=0.01 are marked with a *. The test was only computed for substances for which $f_{censored}$<75%.

| Substance | $f_{censored}$ | Median factor of variation between inlet-outlet samples | Minimum factor of variation between inlet-outlet samples | Maximum factor of variation between inlet-outlet samples |
|---|---|---|---|---|
| $C_{10}$-$C_{12}$ Aliphatics | 0.60 | 1.0 | 1.0 | 14.6 |
| $C_{12}$-$C_{16}$ Aliphatics | 0.50 | 1.0 | 1.2 | 21.7 |
| $C_{16}$-$C_{35}$ Aliphatics | 0.03 | 1.1 | 1.4 | 17.4 |
| $C_{10}$-$C_{16}$ Aromatics | 0.73 | 1.0 | 1.0 | 5.7 |
| Phen | 0.63 | 1.0 | 1.0 | 4.3 |
| Fluo | 0.50 | 1.0 | 1.1 | 8.1 |
| Pyr | 0.50 | 1.0 | 1.0 | 11.5 |
| BaA | 0.63 | 1.0 | 1.0 | 3.1 |
| Chry | 0.60 | 1.0 | 1.1 | 2.8 |
| BbF | 0.53 | 1.0 | 1.1 | 7.7 |
| BaP | 0.67 | 1.0 | 1.0 | 3.9 |
| BPer | 0.57 | 1.0 | 1.1 | 6.2 |
| IP | 0.70 | 1.0 | 1.0 | 4.1 |
| Formaldehyde | 0.03 | 1.0 | 2.0 | 14.5 |
| Acetaldehyde | 0.13 | 1.0 | 1.5 | 5.2 |
| PCB28 | 0.73 | 1.0 | 1.0 | 10.0 |
| PCB52 | 0.47 | 1.0 | 1.1 | 9.6 |
| PCB101 | 0.27 | 1.0 | 1.3 | 21.9 |
| PCB118 | 0.30 | 1.0 | 1.1 | 17.0 |
| PCB153 | 0.27 | 1.0 | 1.2 | 21.7 |
| PCB138 | 0.27 | 1.0 | 1.2 | 22.4 |
| PCB180 | 0.30 | 1.0 | 1.1 | 25.5 |
| PFOS | 0.60 | 1.0 | 1.0 | 4.1 |
| OP | 0.63 | 1.0 | 1.0 | 11.9 |
| NP | 0.63 | 1.0 | 1.0 | 59.7 |
| DBP | 0.70 | 1.0 | 1.0 | 4.0 |
| DEHP | 0.37 | 1.0 | 1.3 | 27.9 |
| DiDP | 0.70 | 1.0 | 1.1 | 6.0 |
| DiNP | 0.67 | 1.0 | 1.5 | 39.1 |
| Monobutyltin | 0.37 | 1.0 | 1.7 | 15.1 |
| Dibutyltin | 0.33 | 1.0 | 1.8 | 35.0 |
| Tributyltin | 0.60 | 1.0 | 1.1 | 13.4 |
| Monooctyltin | 0.57 | 1.0 | 1.1 | 15.5 |
| Dioctyltin | 0.57 | 1.0 | 1.0 | 3.9 |

**Table S10:** Summary of factors of variability within ponds for substances quantified in at least 25% of samples

| Substance | $f_{censored}$ | P-value |
|---|---|---|
| $C_{10}$-$C_{12}$ Aliphatics | 0.60 | 0.390 |
| $C_{12}$-$C_{16}$ Aliphatics | 0.50 | 0.990 |
| $C_{16}$-$C_{35}$ Aliphatics | 0.03 | 0.858 |
| $C_{10}$-$C_{16}$ Aromatics | 0.73 | 0.410 |
| Phen | 0.63 | 0.569 |
| Fluo | 0.50 | 0.638 |
| Pyr | 0.50 | 0.661 |
| BaA | 0.63 | 0.293 |
| Chry | 0.60 | 0.461 |
| BbF | 0.53 | 0.506 |
| BaP | 0.67 | 0.456 |
| BPer | 0.57 | 0.289 |
| IP | 0.70 | 0.661 |
| Formaldehyde | 0.03 | 0.749 |
| Acetaldehyde | 0.13 | 0.608 |
| PCB28 | 0.73 | 0.903 |
| PCB52 | 0.47 | 0.813 |
| PCB101 | 0.27 | 0.981 |
| PCB118 | 0.30 | 0.780 |
| PCB153 | 0.27 | 0.875 |
| PCB138 | 0.27 | 0.931 |
| PCB180 | 0.30 | 0.795 |
| PFOS | 0.60 | 0.322 |
| OP | 0.63 | 0.743 |
| NP | 0.63 | 0.842 |
| DBP | 0.70 | 0.767 |
| DEHP | 0.37 | 0.599 |
| DiDP | 0.70 | 0.756 |
| DiNP | 0.67 | 0.544 |
| Monobutyltin | 0.37 | 0.811 |
| Dibutyltin | 0.33 | 0.892 |
| Tributyltin | 0.60 | 0.609 |
| Monooctyltin | 0.57 | 0.813 |
| Dioctyltin | 0.57 | 0.752 |

**Table S11:** Results of the paired Peto&Peto generalized Wilcoxon test for differences between censored inlet and outlet substance concentrations, n=15 pairs. Significant correlations, considering $\alpha$=0.01 are marked with a * The test was only computed for substances for which $f_{censored}$<75%.

## VII. Photographs of different types of sediments

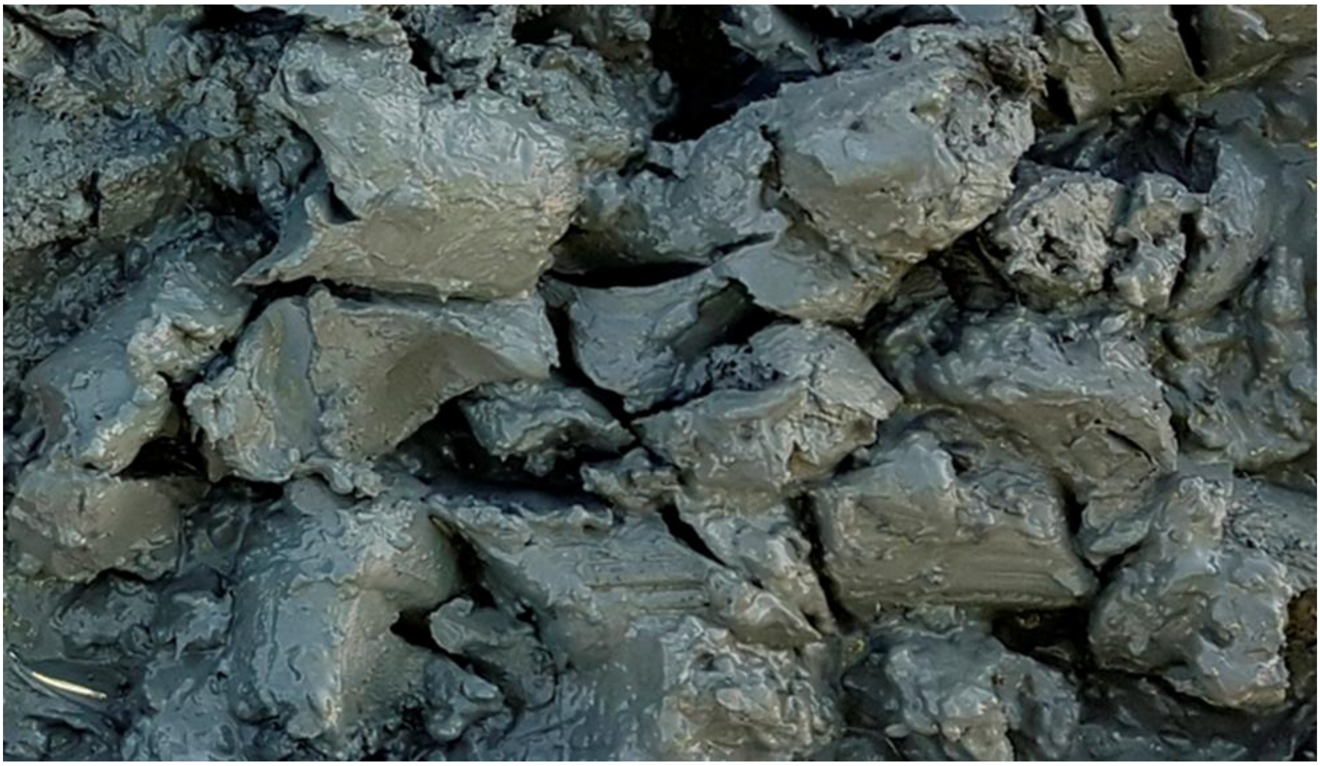

**Figure S1a.** Grey, clay sediments collected from pond Or-2

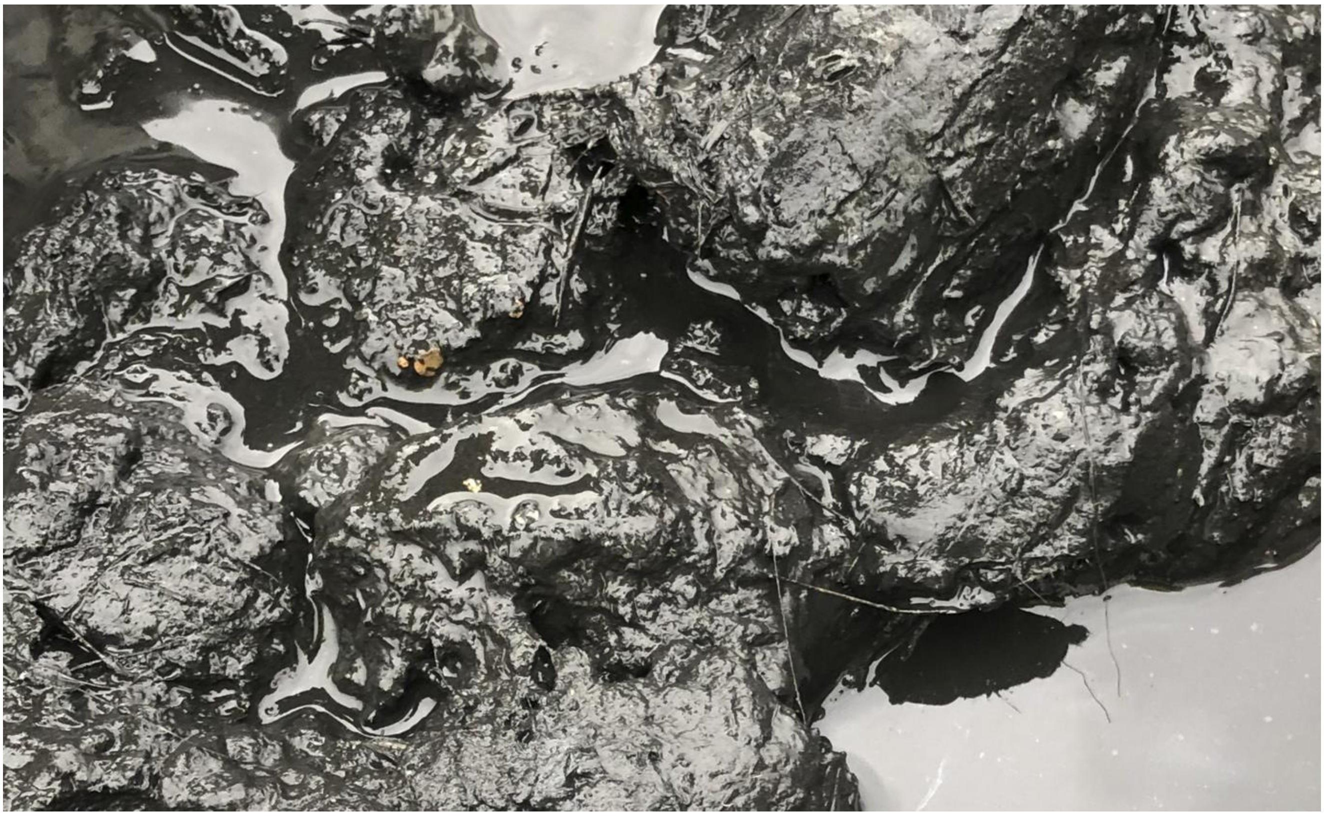

**Figure S1b.** Loose, black sediments collected from pond S-1.

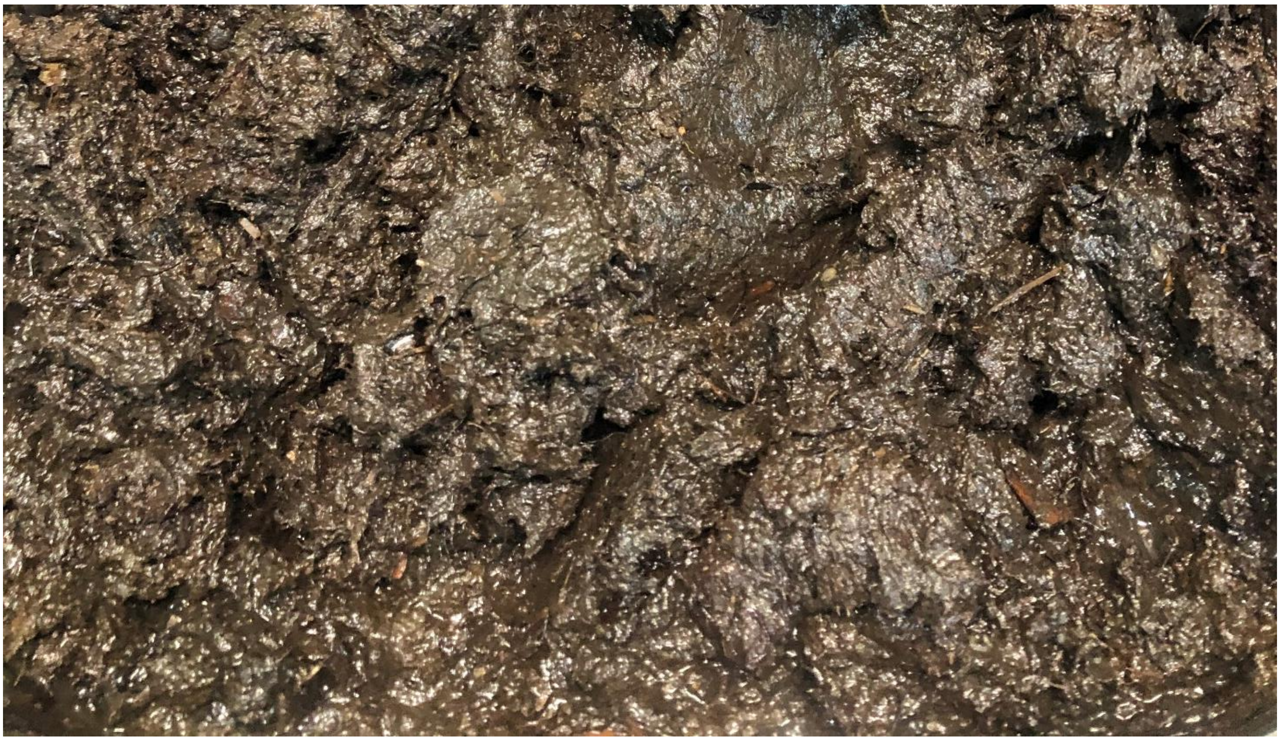

**Figure S3c.** Peat-like sediment collected from pond S-3

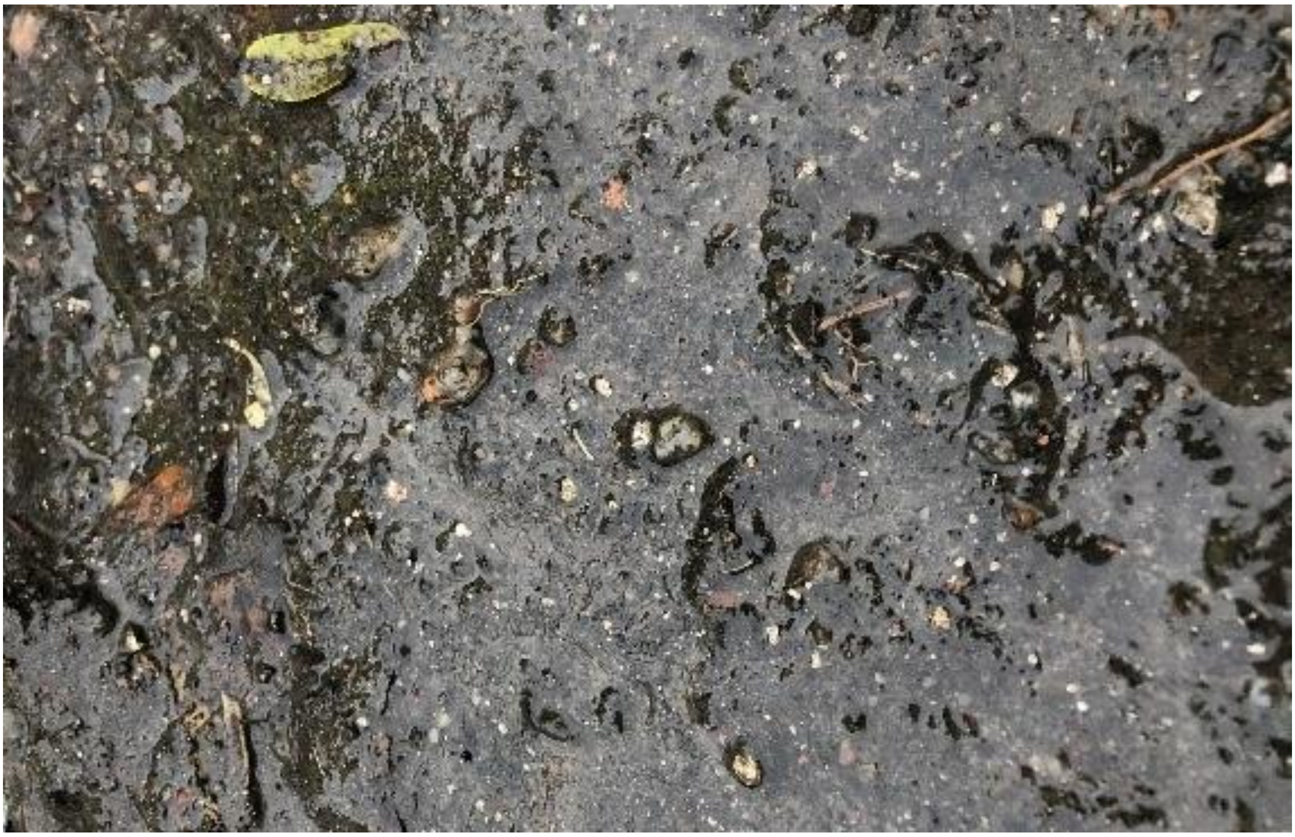

**Figure S1d.** Sandy sediment collected from pond S-5

## VIII. Regulatory limits exceeded by each sample

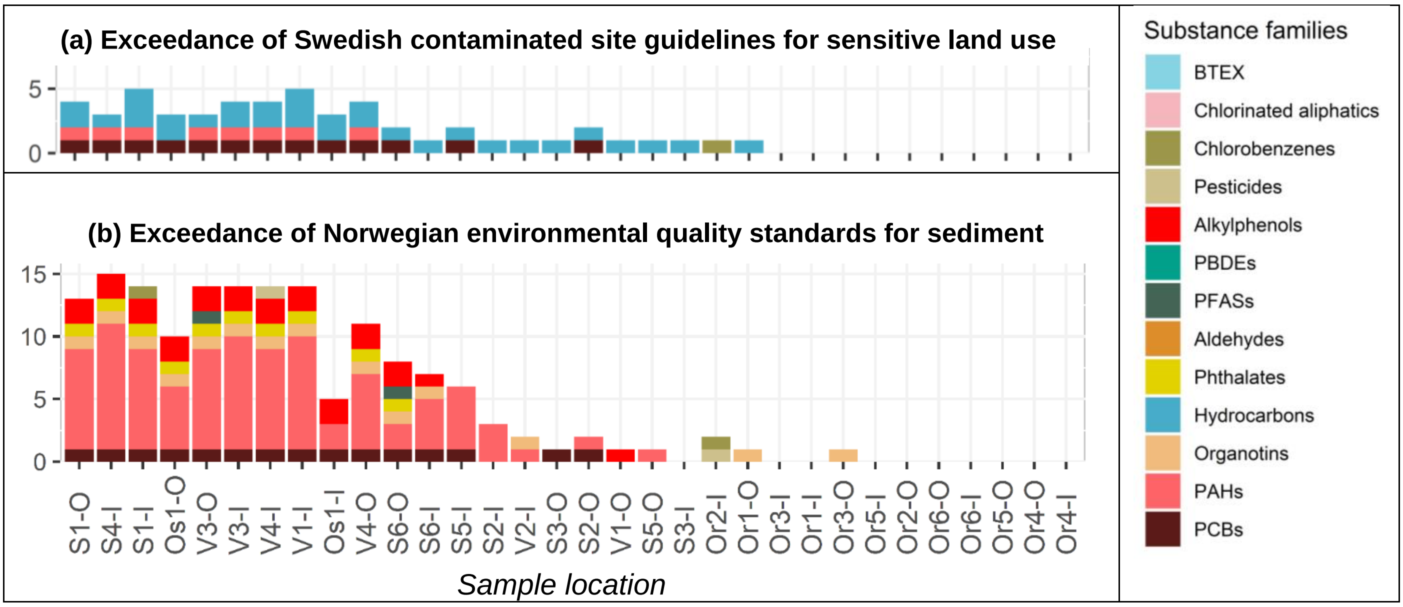


**Figure S2a.** Total number of quantified substances exceeding Swedish contaminated site guidelines for sensitive land use,[50] and **Figure S2b**. total number of quantified substances exceeding Norwegian environmental quality standards for sediment[51]

## IX. Ratios of observed concentrations to regulatory limits

*Note: The following graphs present the ratios of substance concentrations quantified in the stormwater sediments to corresponding environmental standards. Substances below the limit of quantification are not included in this graph. The two standard systems (Swedish contaminated site guidelines and Norwegian environmental quality standards for sediment) do not apply to the same lists of substances; in some cases, standards apply to several substances within a family.*

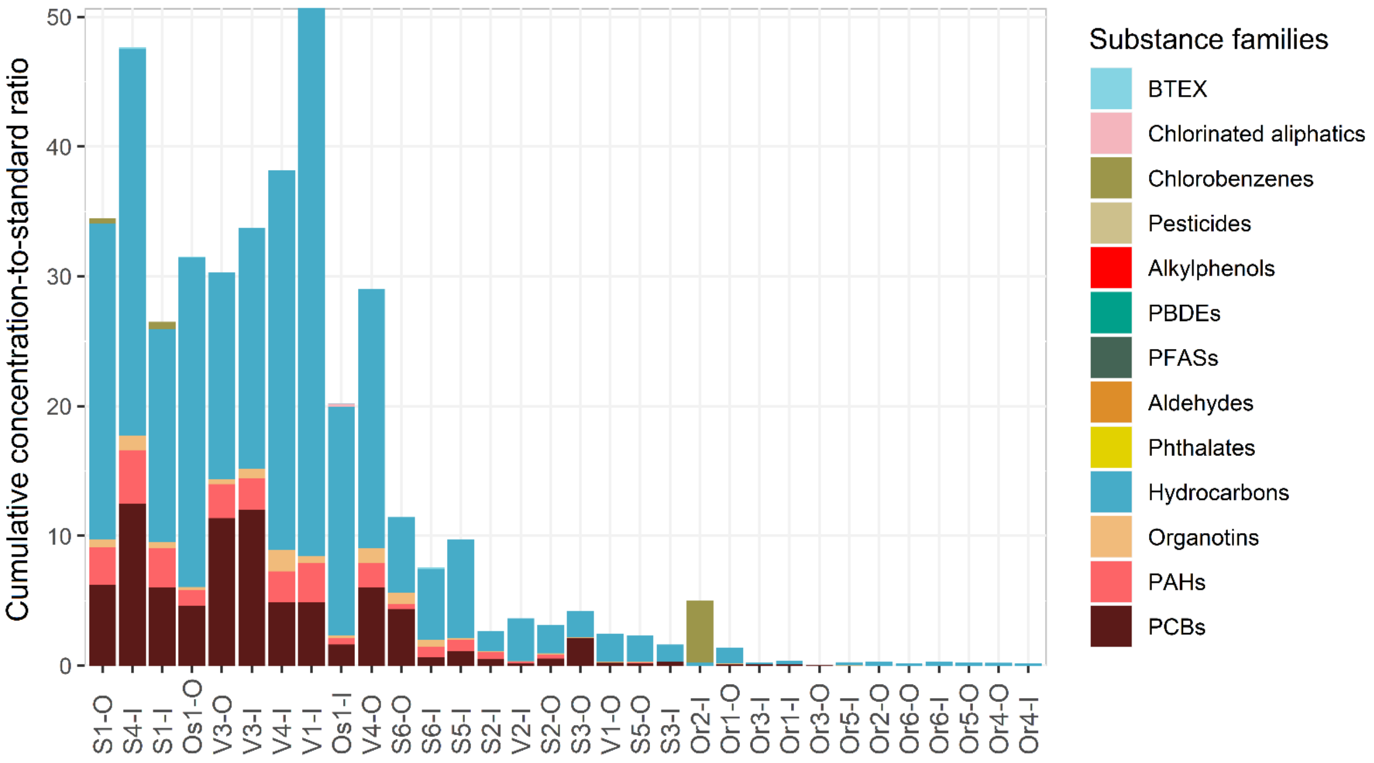


**Figure S3a.** Sum of ratios of quantified substance concentrations to Swedish contaminated site guidelines for sensitive land use,[46] sorted by substance family.

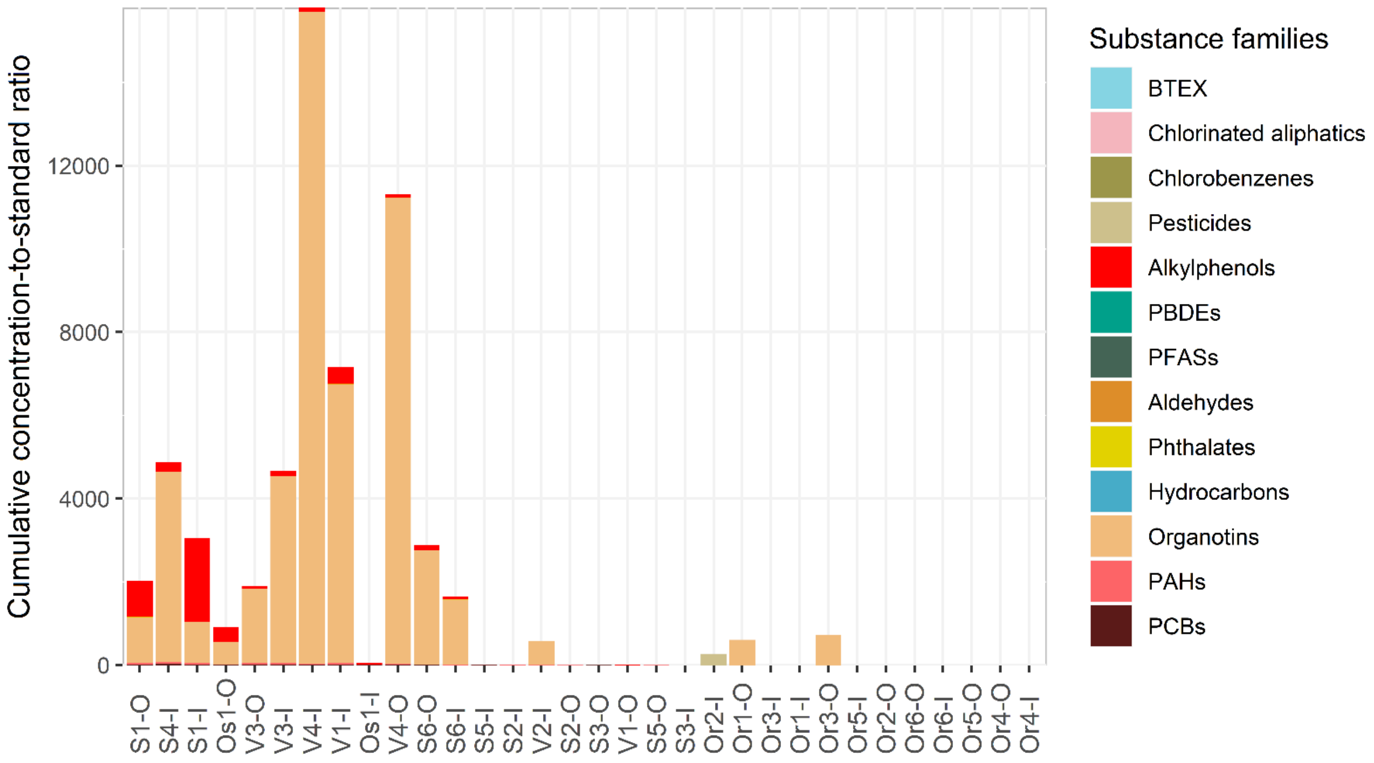


**Figure S3b.** Sum of ratios of quantified substance concentrations to Norwegian environmental quality standards for sediment[47] sorted by substance family.

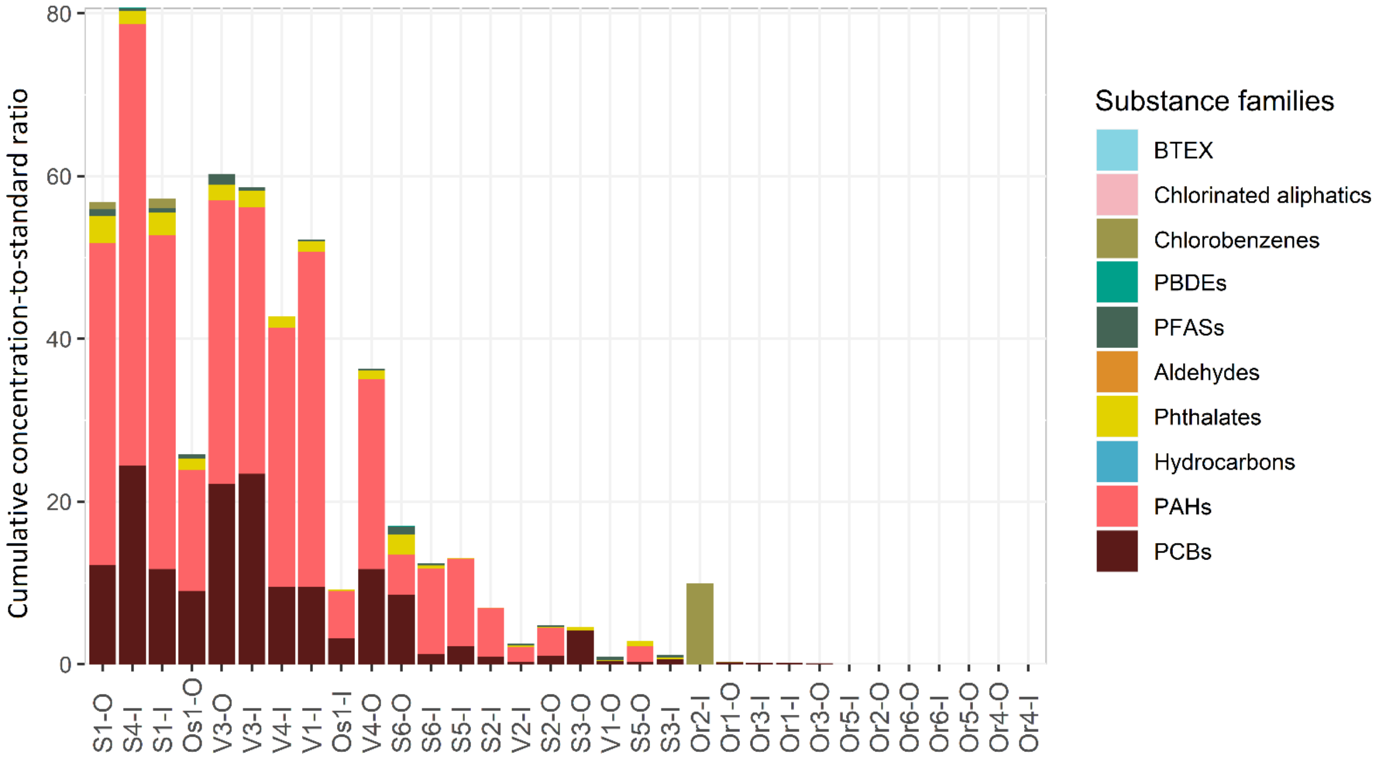


**Figure S3c.** Sum of ratios of quantified substance concentrations to Norwegian environmental quality standards for sediment[47] sorted by substance family, excluding organotins, alkylphenols and pesticides
